# Phollow reveals in situ phage transmission dynamics in the zebrafish gut microbiome at single-virion resolution

**DOI:** 10.1038/s41564-025-01981-1

**Published:** 2025-04-18

**Authors:** Lizett Ortiz de Ora, Elizabeth T. Wiles, Mirjam Zünd, Maria S. Bañuelos, Nancy Haro-Ramirez, Diana S. Suder, Naveena Ujagar, Julio Ayala-Angulo, Calvin Trinh, Courtney Knitter, Shane Gonen, Dequina A. Nicholas, Travis J. Wiles

**Affiliations:** 1 Department of Molecular Biology and Biochemistry, University of California, Irvine, CA USA; 2 Department of Biological Chemistry, School of Medicine, University of California, Irvine, CA USA; 3 Center for Epigenetics and Metabolism, School of Medicine, University of California, Irvine, CA USA; 4 Center for Virus Research, University of California, Irvine, CA USA

**Keywords:** Bacteriophages, Phage biology, Microbiome

## Abstract

Bacteriophages show promise for microbiome engineering, but studying their transmission dynamics in multimember communities and animal hosts is technically challenging. We therefore created ‘Phollow’, a live imaging-based approach for tracking phage replication and spread in situ with single-virion resolution. Following interbacterial phage transmission is achieved by marking virions with distinct fluorescent proteins during assembly in newly infected cells. In vitro cell virology studies revealed clouds of phage virions dispersing upon bacterial lysis, leading to rampant transmission. Combining Phollow with optically transparent zebrafish, we visualized phage outbreaks within the vertebrate gut. We observed that virions from a zebrafish-derived *Plesiomonas* strain, but not a human-derived *E. coli*, rapidly disseminate systemically to the liver and brain. Moreover, antibiotics triggered waves of interbacterial transmission and sudden shifts in gut community ecology. Phollow ultimately empowers multiscale investigations of phage transmission and transkingdom interactions that have the potential to open new avenues for phage-based microbiome therapies.

## Abstract (web summary)

‘Phollow’ is a live imaging-based fluorescence tagging approach that can track phage replication and spread in situ with single-virion resolution.

## Main

Bacteriophages (or ‘phages’) are viruses that infect bacteria. As the most numerous biological entities on Earth, phages exert tremendous influence over microbial communities^1–3^. Phages are largely recognized as lethal predators of bacteria, but they can also enhance bacterial fitness by mediating the transfer of novel genetic traits^4–6^. Identifying how phages shape the form and function of microbiomes could inspire approaches for improving human and environmental health^7–10^. For example, controlled phage outbreaks could be used to deplete disease-causing pathobionts or encourage the spread of beneficial activities. Ultimately, however, harnessing phages to kill or cultivate bacterial communities requires a contextual understanding of phage replication regimes.

Lytic phage replication is a form of horizontal transmission that is overtly antagonistic; phages infect and take over the machinery of bacterial cells to produce new virions, and often disperse through the explosive lysis and death of their host. In contrast, lysogenic replication is a form of vertical transmission that is frequently associated with mutualistic interactions^5,6^. During lysogenic replication, phage genomes are maintained as integrated or episomal prophages that replicate with the bacterial chromosome. Phages capable of forming lysogenic partnerships are referred to as temperate phages.

Through the lens of microbiome engineering, phage replication regimes can be viewed as potential targets for manipulating resident microbial communities. However, the factors governing phage replication and transmission in situ are largely unknown^2,11–13^. This is especially the case within the confines of the gastrointestinal tracts of humans and other animals, where many questions about phage biology remain unanswered. Are outbreaks of lytic replication spatially widespread or locally restricted? Do they occur over rapid or short timescales? What are the consequences of phage replication dynamics on the broader bacterial community, and how might they affect cells and tissues of the animal host? These questions remain largely unexplored because conventional approaches lack spatiotemporal sensitivity and scalable resolution^11,12^. Without knowing the location and duration of phage replication or whether phages are in the form of extracellular virions or intracellular prophages, building a contextualized understanding of phage biology within the gut is severely constrained. We overcome current limitations by creating ‘Phollow’, a collection of tools and techniques for monitoring phage outbreaks in situ by live imaging. Altogether, we show that Phollow is a tractable solution to investigating phage transmission dynamics in the context of microbial communities and living animals.

## Results

### Model phage selection

**Extended Data Fig. 1:**
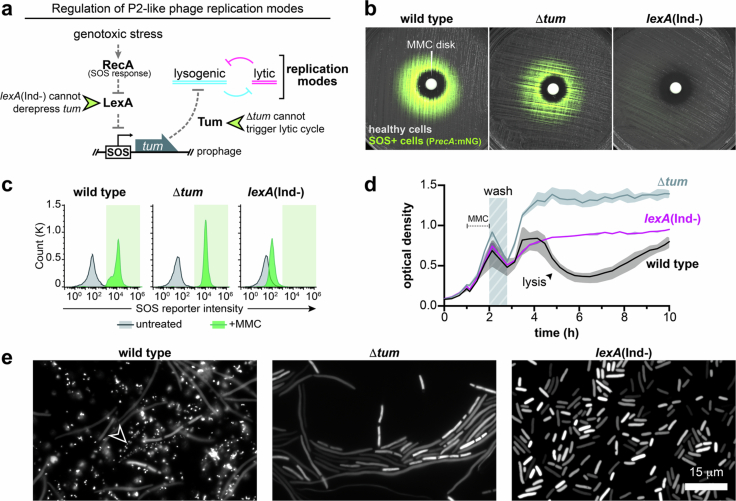
Regulation of P2-like phage DuoHS replication modes. **(a)** Pathway controlling DuoHS replication modes in response to genotoxic stress. DNA damage activates RecA, which mediates the cleavage of the LexA repressor protein. LexA controls the expression of genes within the SOS regulon by binding to operator sequences known as SOS boxes. The LexA-controlled *tum* gene becomes derepressed, which toggles lytic replication by inhibiting pro-lysogenic regulators. Bacterial mutants encoding a non-cleavable *lexA* allele (*lexA*(Ind-)) or lacking *tum* (∆*tum*) are unable to trigger lytic replication. **(b)** Disk diffusion tests visualizing SOS reporter activity across *E. coli* HS strains in response by MMC. MMC generated a zone of inhibition around the assay disk for each strain. However, only the wild-type and ∆*tum* strains displayed SOS reporter activity in cells adjacent to the zone of inhibition. The *lexA*(Ind-) mutant strain did not display SOS reporter activation. **(c)** Quantification of SOS reporter activity following a MMC pulse via flow cytometry. Histograms display the distribution of SOS reporter intensity in untreated (gray) and MMC-treated (green) cultures, with the green shaded area indicating strong reporter activity. The MMC pulse effectively induced SOS reporter activity in wild-type and ∆*tum* strains, but not the *lexA*(Ind-) mutant. **(d)** Representative induction (or ‘lysis’) curve of DuoHS lytic replication in *E. coli* HS. The optical density of cultures (at 600 nm) containing either wild-type (black line), ∆*tum* (gray line) or *lexA*(Ind-) (purple line) are shown. Lines represent the average and shaded regions represent the minimum and maximum from three technical replicates. MMC was washed from cultures at 2 h (hashed bar), which prevents interference of phage lytic replication. An abrupt drop in optical density of the wild-type culture at 4 h marks widespread lysis, which is not observed in ∆*tum* or *lexA*(Ind-) cultures. **(e)** Fluorescence microscopy images of wild-type, ∆*tum*, and *lexA*(Ind-) Phollow virocell strains 1 h post-MMC treatment. Wild-type cells show extensive filamentation and formation of viral foci (arrowhead), which are not displayed by ∆*tum* and *lexA*(Ind-) mutant strains. *lexA*(Ind-) cells do not exhibit cell filamentation due to an impaired SOS response.

We selected P2-like phages as a model system for developing approaches to visualize phage replication regimes. P2-like phages belong to the Caudoviricetes class of tailed phages and infect over 120 bacterial genera across the phylum Proteobacteria^14–16^. P2-like phages display a temperate lifestyle, which enables the study of both lytic and lysogenic replication. Lytic replication of the P2-like phages used in our study is induced by DNA damage and activation of the ‘SOS’ response (Extended Data Fig. 1).

### Design, construction and infectivity of Phollow phages

We devised an in vivo tagging system in which phage virions are fluorescently marked during intracellular assembly, but then become marked by a different colour upon infection and replication within a new bacterial host. This combinatorial labelling strategy, which we call ‘Phollow’, makes it possible to ‘follow’ interbacterial transmission during phage outbreaks. We have provided a guide to implementing Phollow that outlines technical considerations and lessons learned in Supplementary Information.

**Fig. 1:**
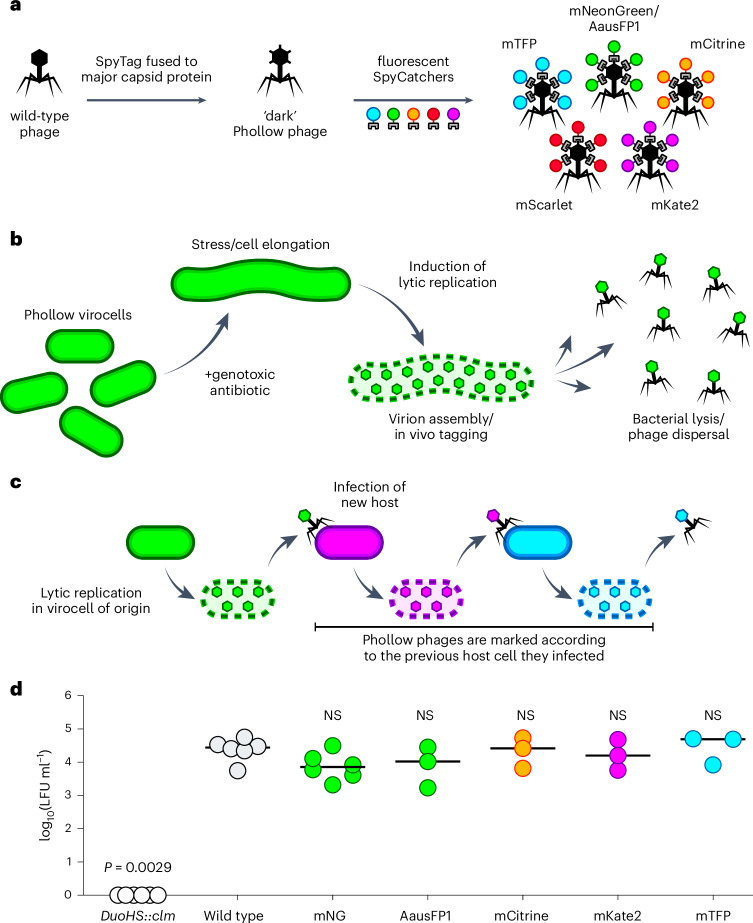
Design, construction and infectivity of Phollow phages. **a**, Design overview of Phollow phages using the SpyTag:SpyCatcher system. **b**, Cartoon schematic of phage tagging in vivo using Phollow. Phollow virocells treated with genotoxic antibiotics to induce lytic replication display hallmark morphological changes upon activation of the DNA damage ‘SOS’ response, namely, cell filamentation. As phage gene activation and replication proceed, fluorescent SpyCatcher proteins redistribute from the cytosol to sites of capsid assembly. Upon lysis, fluorescently tagged Phollow phage virions are released. **c**, Cartoon schematic of combinatorial Phollow tagging to track interbacterial phage transmission. **d**, Phollow phages display similar levels of infectivity compared to a wild-type DuoHS phage. The infectivity of wild-type and differentially marked Phollow phages was assessed by measuring lysogen-forming units (LFUs) generated from infection of an *E. coli* target cell. Lysogeny was monitored using prophages carrying a chloramphenicol resistance gene. *DuoHS::clm* is an *E. coli* HS strain that carries a chloramphenicol resistance marker in place of the DuoHS prophage genome and is used to control for phage-independent transfer of chloramphenicol resistance. Bars denote medians and circles represent independent biological replicates (*DuoHS::clm*, wild type, mNG: *n* = 6 each; AausFP1, mCitrine, mKate2, mTFP: *n* = 3 each). Significant differences compared to ‘wild type’ were determined using Kruskal–Wallis and Dunn’s multiple comparisons test (*P* < 0.05; NS, not significant).

**Extended Data Fig. 2:**
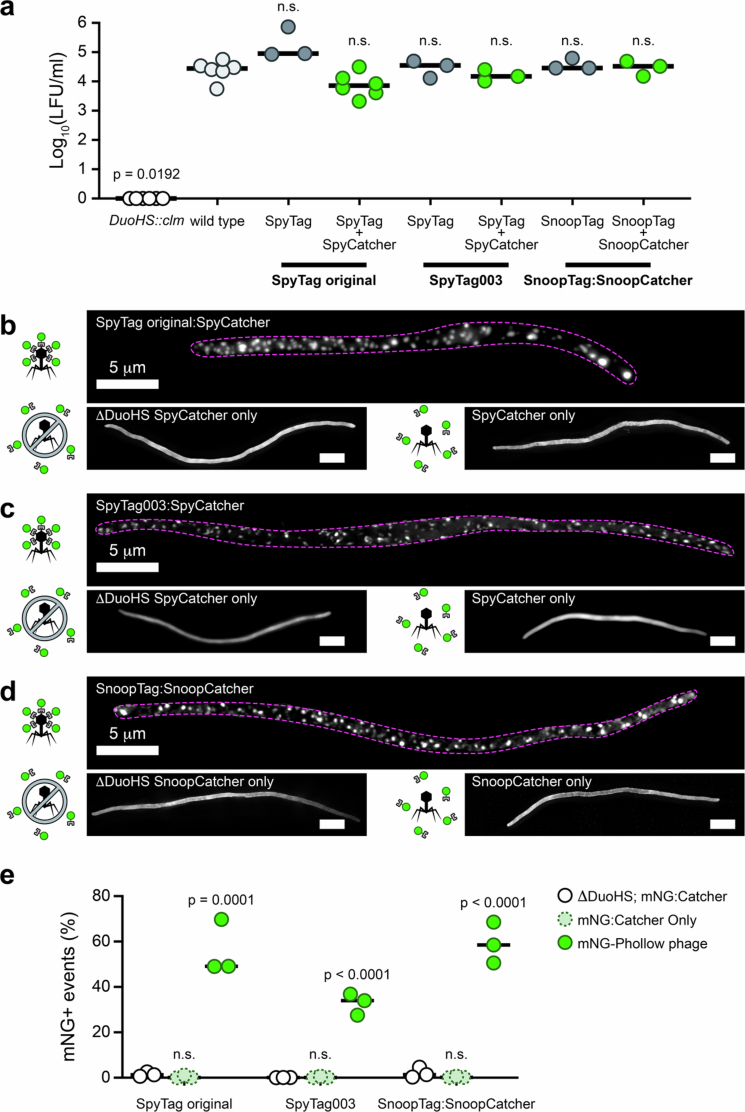
Characterization of Phollow phage tagging variants. **(a)** The infectivity of wild-type and different Phollow phage variants was assessed by measuring lysogen-forming units (LFU) generated from infection of an *E. coli* HS target cell cured of its resident DuoHS prophage. Lysogeny was monitored using prophages carrying a chloramphenicol resistance gene. *DuoHS::clm* is an *E. coli* HS strain that carries a chloramphenicol resistance marker in place of the DuoHS prophage genome and is used to control for phage-independent transfer of chloramphenicol resistance. Bars denote medians and each circle represents an independent biological replicate. Significant differences compared to ‘wild type’ were determined using a Kruskal-Wallis and Dunn’s multiple comparisons test (p < 0.05; n.s. = not significant). **(b-d)** Comparison of viral foci production and organization across Phollow phage variants: (**b**) SpyTag original:SpyCatcher; (**c**) SpyTag0003:SpyCatcher; (**d**) SnoopTag:SnoopCatcher. The top of each panel shows a representative maximum intensity projection image of viral foci organization within Phollow virocells capable of generating fully tagged Phollow phages (a dashed magenta line highlights the cell perimeter). The bottom left of each panel shows a representative maximum intensity projection image of Spy- or SnoopCatcher-based fluorescence in the absence of a DuoHS prophage (∆DuoHS). The bottom right of each panel shows a representative maximum intensity projection image of Spy- or SnoopCatcher-based fluorescence in the presence of a lytically replicating DuoHS that lacks a Spy- or SnoopTag, which is thus unlabeled. No viral foci form in the absence of a fully tagged Phollow phage. **(e)** Plot shows the functionality of each Phollow phage variant to be used for flow virometry. Data are presented as the percentage of mNeonGreen (mNG) positive events. Bars denote medians and statistical differences compared to the ‘∆DuoHS; mNG:catcher’ control strain within each variant group were determined by an ordinary one-way ANOVA using a Dunn’s multiple comparisons test (SpyTag original: p = 0.0001; SpyTag003: p = 0.0001; SnoopTag: p = 0.0001; n.s. = not significant; 3 biological replicates are shown for each condition).

We constructed fluorescently marked ‘Phollow phages’ using the SpyTag:SpyCatcher tagging system^17,18^ (Fig. 1a and Supplementary Data 1). The major capsid protein (GpN) of a P2-like prophage (which we have named ‘DuoHS’) harboured within *Escherichia coli* HS^19,20^ (a human gut-derived commensal) was modified to contain a C-terminal peptide linker and SpyTag. We inserted a Tn*7* transposon into the bacterial chromosome carrying a constitutively expressed gene encoding SpyCatcher fused to one of several distinct fluorescent proteins. SpyTags decorating phage capsids become covalently bound by SpyCatcher proteins during virion assembly, generating fluorescently marked Phollow phages (Fig. 1a). We refer to engineered bacterial strains capable of producing Phollow phage virions as ‘Phollow virocells’. We note that we also assessed the functionality of other SpyTag variants^21^ and the SnoopTag:SnoopCatcher system^22^ (Extended Data Fig. 2 and Supplementary Data 1).

As designed, SpyCatcher proteins redistribute from the bacterial cytosol to assembling Phollow phage capsids during lytic replication (Fig. 1b). Host lysis then leads to the dispersal of fluorescently marked Phollow phage virions throughout the environment (Fig. 1b). Tracking interbacterial transmission is accomplished using target bacterial cells expressing SpyCatcher variants that have a different colour from those expressed by the original Phollow virocell. Infection of differentially marked bacterial hosts, therefore, produces Phollow phages donning new fluorescent tags (Fig. 1c). Phollow phages exhibit similar infectivity compared to a non-fluorescently tagged phage, emphasizing their capacity to recapitulate wild-type replication and transmission (Fig. 1d).

### Investigating the cell biology of phage lytic replication

**Fig. 2:**
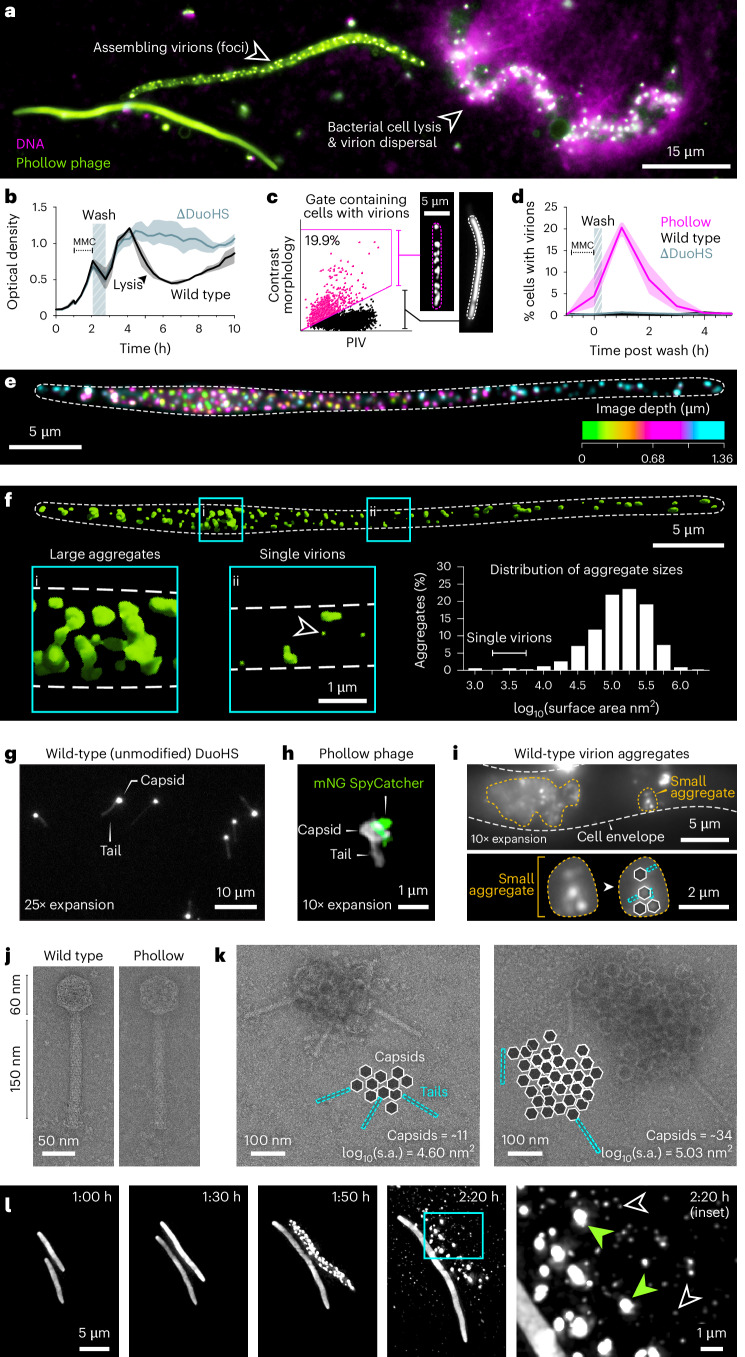
Investigating the cell biology of phage lytic replication. **a**, *E. coli* Phollow virocells in different phases of lytic replication: cell filamentation (left), virion assembly (middle), and cell lysis and release of viral particles (right). DNA from the lysing cell is labelled with the cell-impermeable DNA dye EthD-III (magenta). **b**, Representative induction curve of DuoHS phage lytic replication in *E. coli* HS. Shown are changes in optical density of cultures containing wild-type (black line) or a DuoHS prophage-cured strain (∆DuoHS, grey line). Lines represent averages and shaded regions represent the minimum and maximum of 3 technical replicates. **c**, Imaging flow cytometry gating scheme for quantifying cells harbouring lytically replicating Phollow phages (marked by magenta box and hashed line). PIV, pixel intensity variance. **d**, Imaging flow cytometry-based quantification of lytic replication over time. Lines represent averages and shaded regions represent minimum and maximum of 3 biological replicates. **e**, *Z*-projected image of a bacterial cell harbouring viral foci, pseudo-coloured according to *z*-depth. White dashed line marks the cell perimeter. **f**, 3D rendering of the cell shown in **e**. (**i**) large virion aggregates. (**ii**) single virions (arrowhead). Histogram shows aggregate sizes based on 2D surface areas (*n* = 357 aggregates from 5 cells). **g**, ExM image of unmodified wild-type DuoHS virions. **h**, ExM image of a DuoHS Phollow virion labelled with an mNeonGreen (mNG) SpyCatcher peptide. **i**, Top: ExM image of an *E. coli* cell containing unmodified lytically replicating wild-type DuoHS phage. Amber dashed outlines indicate virion aggregates. Bottom: an enlarged image of the small virion aggregate, highlighting virion morphologies. **j**, Representative TEM micrographs of unmodified wild-type (left) and Phollow phage (right) virions. This experiment was independently replicated at least twice with similar results. **k**, TEM micrographs of 2 example extracellular virion aggregates of unmodified wild-type DuoHS phages. Cartoons diagram capsid and tail components within each aggregate. The surface area (s.a.) of each aggregate was estimated on the basis of the area encompassing discernible capsid structures. This experiment was independently replicated at least twice with similar results. **l**, Time-lapse images showing the induction, assembly and dispersal of virions and aggregates upon cell lysis. Right inset: black arrowheads mark single virions and small aggregates; green arrowheads mark large virion aggregates.

**Extended Data Fig. 3:**
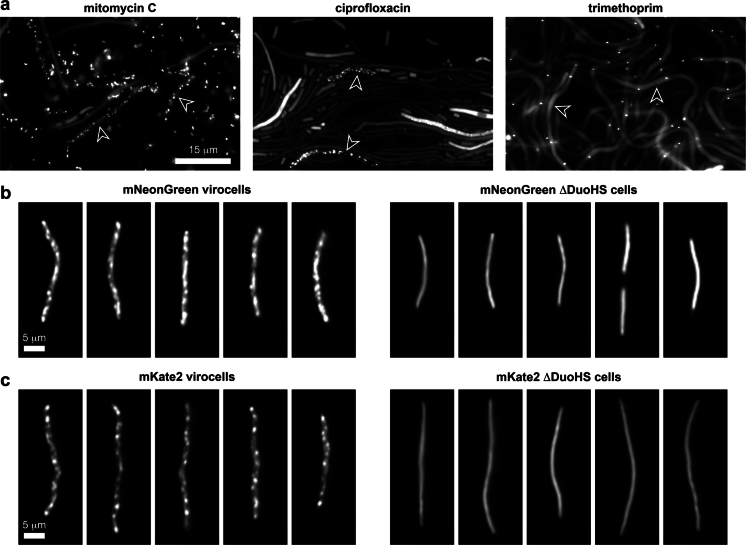
Genotoxic antibiotics induce similar but distinct patterns of bacterial cell filamentation and viral foci production. **(a)** Fluorescence microscopy images of mNeonGreen *E. coli* Phollow virocells treated with (left) mitomycin C, (middle) ciprofloxacin, or (right) trimethoprim. Widespread cell filamentation, a common response to genotoxic antibiotics, is seen in each condition along with the production of viral foci. However, each antibiotic induces different degrees of filamentation and frequencies of viral foci formation. Arrowheads in each image indicate examples of filamented cells containing viral foci. The image shown for mitomycin C was taken from a different field of view of the sample shown in Extended Data Fig. 1e ‘wild type’, and is shown for comparison. **(b,c)** Example flow cytometry images of (**b**) mNeonGreen or (**c**) mKate2 virocells containing viral foci induced by mitomycin C. Shown on the right of each panel are example flow cytometry images of ΔDuoHS control cells expressing each respective fluorescent Catcher peptide, which have also been treated with mitomycin C.

We first applied Phollow to probe intracellular features of P2-like phage lytic replication. Remarkably, we captured the complete sequence of Phollow phage assembly and dispersal within a single field of view (Fig. 2a). Treating *E. coli* Phollow virocells with the DNA-damaging agent mitomycin C (MMC) led to cellular filamentation and the formation of fluorescent viral foci, culminating in bacterial lysis and the extracellular release of virions. Because antibiotics have wide-ranging physiological effects on bacterial cells that could alter phage induction and virion assembly, we tested two additional genotoxic drugs: ciprofloxacin and trimethoprim. We found that each antibiotic induced cellular filamentation and viral foci like MMC; however, the degree of filamentation and number of viral foci per cell was antibiotic specific (Extended Data Fig. 3a). Leveraging the cell virological traits of lytically replicating Phollow phages, we used imaging flow cytometry to quantify MMC induction kinetics, spanning initial treatment to bacterial lysis (Fig. 2b). Gating on features of cell filamentation and the presence of viral foci (Fig. 2c and Extended Data Fig. 3b, c), we found that peak induction occurred at 1 h post-MMC treatment and comprised ~20% of the bacterial population (Fig. 2d). The number of cells with lytically replicating phage fell sharply over time, corresponding to cell lysis (Fig. 2b,d).

**Extended Data Fig. 4:**
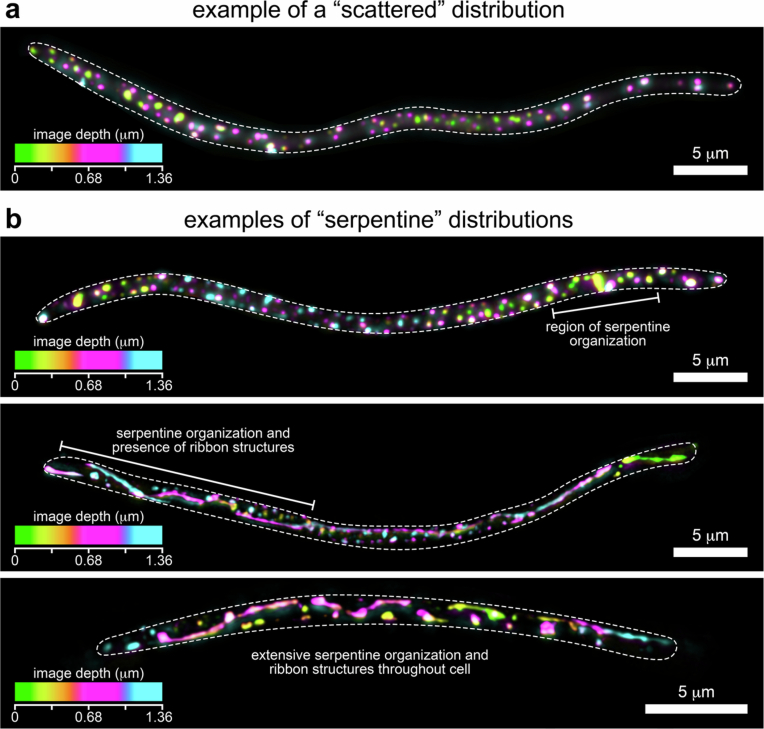
Examples of intracellular Phollow phage organization. **(a-b)** Z-projected images of bacterial cells harboring viral foci. Images highlight two patterns of virion organization throughout the intracellular space: (**a**) a scattered distribution and (**b**) a serpentine pattern with the presence of ribbon structures. Images are pseudo-colored according to z-depth, representing a total of 1.36 μm. A white dashed line marks the cell perimeter.

**Extended Data Fig. 5:**
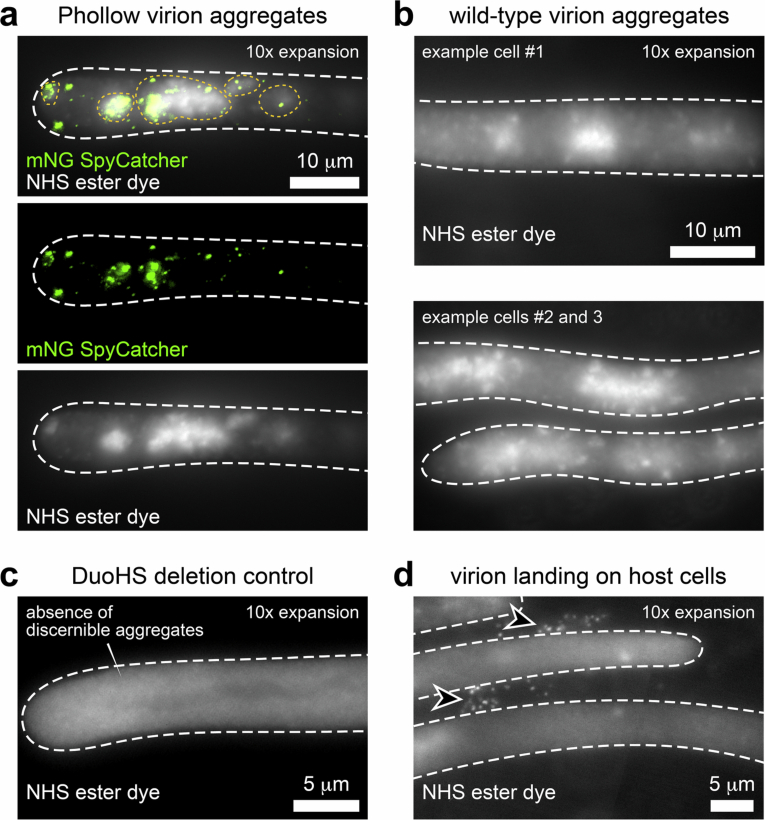
Expansion microscopy (ExM) of intracellular virion aggregates. **(a)** ExM image showing a single *E. coli* Phollow virocell containing lytically replicating mNeonGreen (mNG) Phollow phage. A white dashed line in each image marks the bacterial cell envelope. The top image is a merge of the mNG (phage capsid, green) and N-Hydroxysuccinimide (NHS) ester dye (protein, grayscale) channels. The middle and bottom images show each individual channel. Amber dashed outlines in the top image indicate putative multi-virion aggregates. **(b)** ExM images showing additional examples of *E. coli* cells containing lytically replicating unmodified wild-type DuoHS phage. White dashed lines in each image mark the bacterial cell envelope. **(c)** ExM image showing an *E. coli* cell in which the DuoHS prophage has been deleted. The cell was treated with mitomycin C to simulate induction of lytic replication. The cell does not display virion aggregate-like structures. A white dashed line marks the bacterial cell envelope. **(d)** ExM image showing unmodified wild-type DuoHS virions landing on the surface of *E. coli* cells. White dashed lines mark the bacterial cell envelope.

**Extended Data Fig. 6:**
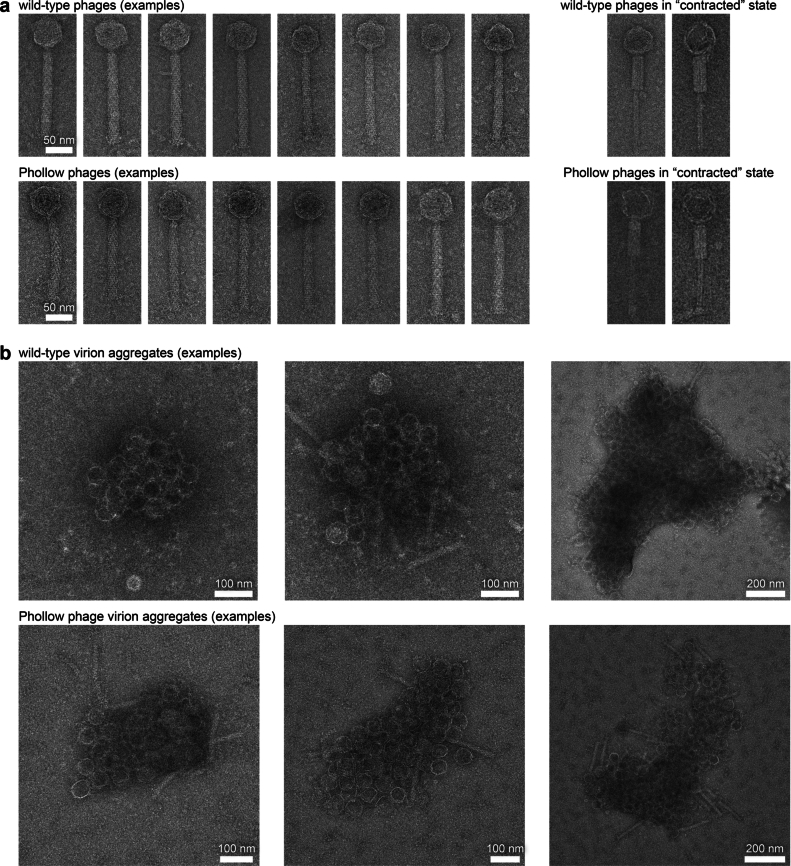
Transmission electron microscopy (TEM) of negatively stained DuoHS virions and virion aggregates. **(a)** Representative TEM micrographs of unmodified wild-type (top) and mNeonGreen Phollow phage virions (bottom). Also included are example virions of each phage with contracted tail sheaths, which is further evidence that Phollow phage virions are functional and competent for infection. Experiment was independently replicated at least twice with similar results. **(b)** TEM micrographs of extracellular virion aggregates from unmodified wild-type DuoHS (top) and mNeonGreen Phollow phage (bottom). Experiment was independently replicated at least twice with similar results.

At peak lytic replication, we estimate that Phollow virocells harbour an average of 1.6 ± 0.4 viral foci per micron cell length (mean length = 44.92 ± 7.11 μm, *n* = 5) (Fig. 2e). Viral foci most often displayed a scattered distribution; however, we also observed serpentine patterns that could indicate early organizational dynamics of capsid proteins (Supplementary Movie 1 and Extended Data Fig. 4). Three-dimensional (3D) projections of viral foci revealed that they have an average surface area that is ~100 times larger than a single P2-like phage capsid with a diameter of 60 nm (ref. ^14^), suggesting that they represent multivirion aggregates (Fig. 2f). To better resolve multivirion structures, we performed expansion microscopy (ExM). ExM made it possible to image fully assembled virions of both unmodified wild-type DuoHS and its Phollow phage derivative (Fig. 2g,h). We found that wild-type DuoHS also formed aggregate structures of varying sizes, with distinct virion morphologies being clearly visible in some instances (Fig. 2i and Extended Data Fig. 5). Comparing unmodified wild-type and Phollow phage virions by transmission electron microscopy (TEM), we did not find evidence of overt structural differences between the two (Fig. 2j and Extended Data Fig. 6a). As with ExM, TEM revealed that aggregates generated by wild-type DuoHS phages ranged in size similar to those from Phollow phages identified by fluorescence microscopy (Fig. 2k and Extended Data Fig. 6b).

To ascertain the fate of virion aggregates, we performed time-lapse imaging of Phollow phage virion assembly and dispersal. At 2 h post induction, aggregates began to form within cells, followed by widespread bacterial lysis shortly after (Fig. 2l and Supplementary Movie 2). Aggregates quickly dispersed into clouds of rapidly diffusing particles, suggesting that bacterial lysis and viral disaggregation are coordinated (Fig. 2l, 2:20 h inset). Altogether, these experiments demonstrate the utility of Phollow for studies of cellular virology, from intracellular induction and virion assembly to bacterial lysis and dispersal.

### Monitoring virion dispersal by flow virometry

**Fig. 3:**
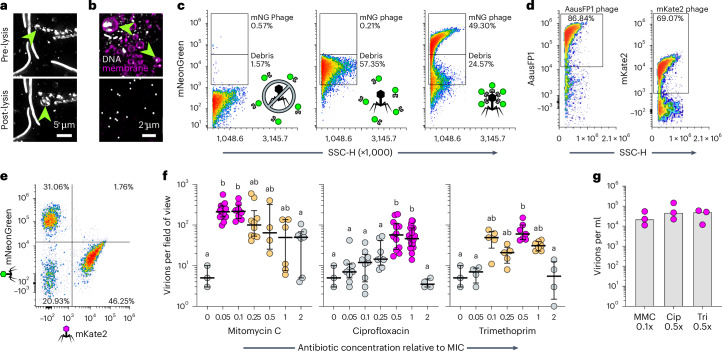
Monitoring virion dispersal by fluorescence microscopy and flow virometry. **a**, Maximum intensity projection image of MMC-induced Phollow virocells before (top) and after a lysis (bottom). Green arrowheads indicate cells that give rise to membrane vesicles containing virion aggregates and cytosolic SpyCatcher protein. **b**, Top: MMC-induced cultures of *E. coli* carrying ‘dark’ Phollow phages. Membrane and DNA staining reveals numerous membrane vesicles that frequently contain DNA (green arrowheads). Bottom: purified lysates show fewer vesicles and contain discernible virion-sized DNA-positive puncta. **c**, Gating strategy for quantifying Phollow phage virions in purified lysates by flow virometry. **d**, Representative flow virometry plots showing gates for AausFP1 (left) and mKate2 (right) Phollow phage virions. **e**, Representative flow virometry plot showing the quantification of Phollow phage virions from a mixed lysate. **f**, Fluorescence microscopy-based quantification of Phollow phage virion production in response to treatment with the genotoxic antibiotics mitomycin C (left), ciprofloxacin (middle) or trimethoprim (right). Antibiotic concentrations are given relative to each antibiotic’s MIC against wild-type *E. coli* HS. Bars indicate medians and interquartile ranges. Circles represent distinct and non-overlapping fields of view; data were pooled from 3 biological replicates. Data for the ‘0’ concentration is the same for all plots and sets a statistical baseline. Statistical groupings (denoted by letters and colour coding) in each plot were determined using Kruskal–Wallis and Dunn’s multiple comparisons test (*P* *=* 0.0001). **g**, Flow virometry-based quantification of Phollow phage virion production induced by mitomycin C (MMC, 0.1× MIC), ciprofloxacin (Cip, 0.5× MIC) or trimethoprim (Tri, 0.5× MIC). Bars indicate medians derived from 3 biological replicates (circles). No statistical differences were found using Kruskal–Wallis and Dunn’s multiple comparisons test (*P* *=* 0.8286).

Quantifying extracellular virions can be used to estimate the potential for horizontal transmission. However, differentiating viral particles from cellular debris and vesicles is challenging with high-sensitivity imaging and flow cytometry techniques^23^. Underscoring this problem, many vesicles produced during lytic replication contain cytosolic contents (including virions) and DNA (Fig. 3a,b). Implementing established purification steps^24^ facilitated straightforward identification of virions by microscopy (Fig. 3b, bottom), but the residual debris in viral preparations continued to interfere with flow cytometric analysis. We therefore utilized Phollow virocell control strains to develop a flow virometry gating strategy to differentiate free viral particles from cell debris. We discerned cell debris originating from bacteria expressing a fluorescent SpyCatcher protein but not carrying a DuoHS prophage (Fig. 3c, left). We then identified fluorescent debris and vesicles associated with lytic replication using bacteria expressing a fluorescent SpyCatcher protein and carrying an unmodified wild-type prophage (Fig. 3c, middle). With these gates established, we could confidently quantify fluorescently marked Phollow phages (Fig. 3c, right). We found that similar gating strategies can be used for additional fluorescent proteins (Fig. 3d), and it is possible to parse mixed populations of differentially marked Phollow phages (Fig. 3e).

**Extended Data Fig. 7:**
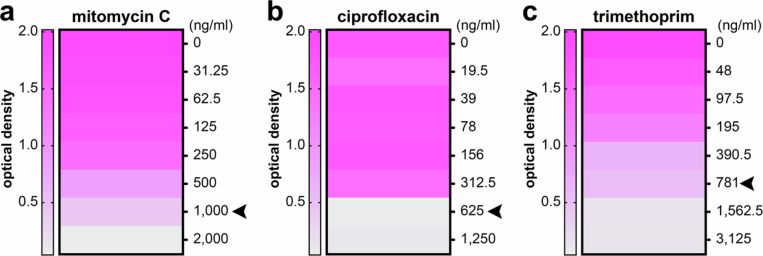
Determination of minimum inhibitory concentrations of genotoxic antibiotics for *E. coli* HS. **(a-c)** Optical density at 600 nm measurements of *E. coli* HS cultures in response to various concentrations of (**a**) MMC, (**b**) ciprofloxacin, and (**c**) trimethoprim. Data represent the average of three independent endpoint measurements after 24 hours of incubation at 37 °C, plotted as heatmaps. Arrowheads in each panel indicate the minimum inhibitory concentration (MIC), defined as the minimum concentration required to significantly impair bacterial growth without complete eradication.

We utilized Phollow to carry out a flow virometry-based study comparing the magnitude of virion dispersal induced by genotoxic antibiotics with different modes of action and minimum inhibitory concentrations (MIC) (Extended Data Fig. 7). Screening virion production by fluorescence microscopy (Fig. 3f) first revealed that MMC, a DNA crosslinking agent, displays potent activity over a broad concentration range. In contrast, the DNA gyrase inhibitor ciprofloxacin induces the production of viral particles across a narrow concentration range. Lastly, trimethoprim, which interferes with thymidine synthesis, induces virion production over an intermediate concentration range. Flow virometry revealed that peak virion output across the three antibiotics was essentially equal, suggesting that they have similar capacities for spurring lytic replication despite their unique induction profiles (Fig. 3g). Together, this survey of antibiotic-specific induction activities demonstrates how Phollow expedites flow virometry-based studies capable of uncovering contextual modulators of virion dispersal.

### Visualizing phage outbreaks within the vertebrate gut

**Fig. 4:**
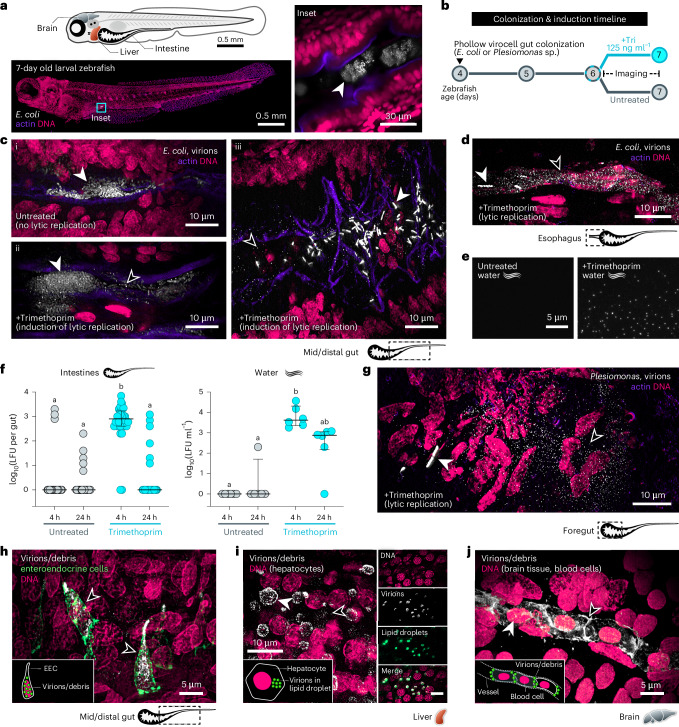
Visualizing outbreaks of phage lytic replication within the vertebrate gut. **a**, Top: larval zebrafish schematic. Bottom: maximum intensity projection of a larval zebrafish colonized by *E. coli*. Inset: image showing luminal *E. coli* aggregates (arrowhead). DNA (magenta) and actin (purple) highlight intestinal structure. **b**, Bacterial colonization and antibiotic induction timeline. ‘Tri’, trimethoprim. **c**, (**i**) Maximum intensity projection image of untreated *E. coli* Phollow virocells within the gut (white arrowhead). (**ii**,**iii**) Maximum intensity projection images of trimethoprim-treated *E. coli* Phollow virocell populations. White arrowheads mark bacterial aggregates and single cells; black arrowheads mark viral particles. **d**, Maximum intensity projection image showing viral particles in the oesophageal region. White arrowhead marks a single cell; black arrowhead marks viral particles. **e**, Fluorescence microscopy images of water samples from untreated (left) or trimethoprim-treated (right) zebrafish. **f**, Quantification of infectious virions from intestinal tissues (left) and water (right) post-trimethoprim treatment (cyan circles). Infectious virions from untreated samples are in grey. Bars indicate medians and interquartile ranges. Statistical groupings (denoted by letters) determined using Kruskal–Wallis and Dunn’s multiple comparisons test (intestines: *P* = 0.0001; water: *P* = 0.0002). **g**, Maximum intensity projection image showing viral particles derived from *Plesiomonas* Phollow virocells within the intestine. White arrowhead marks a single cell; black arrowhead marks viral particles. **h**, Maximum intensity projection image showing viral particles within enteroendocrine cells (EEC, green). Black arrowheads mark EECs containing viral particles or debris. Inset: cartoon representation of viral particles associating with an EEC. **i**, Left: maximum intensity projection image showing viral particles associating with hepatocytes. White arrowhead marks virions within a vesicle-like structure; black arrowhead marks a virion aggregate or single phage. Inset: cartoon representation of viral particles associating with hepatocytes. Right: montage shows liver tissue from a separate fish stained to highlight lipid droplets (green). **j**, Maximum intensity projection image showing viral particles within a blood vessel in the brain. White arrowhead marks a nucleated red blood cell; black arrowhead marks virions or phage debris associating with the surface of blood cells. Inset: cartoon representation of viral particles associating with red blood cells.

**Extended Data Fig. 8:**
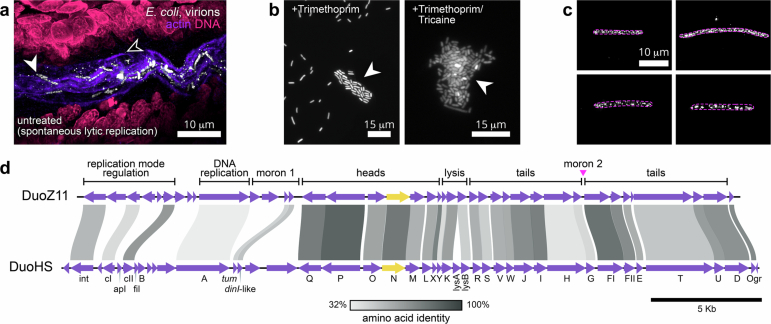
Characterization of phage outbreaks within the vertebrate gut. **(a)** Maximum intensity projection image showing viral particles derived from untreated *E. coli* HS Phollow virocells undergoing spontaneous phage lytic replication within the intestine. White arrowhead marks bacterial cells; black arrowhead marks viral particles. **(b)** Representative fluorescent microscopy images of AausFP1 *E. coli* HS Phollow virocell in zebrafish embryo media (EM) in the absence of fish. Bacteria were separated from fish immediately before the experiment to allow for the diffusion of fish-derived nutrients and other factors. Subsequently, bacteria were treated with trimethoprim (left) or trimethoprim and tricaine (right) and incubated at 28.5 °C for 4 h. Bacterial cells under these conditions do not display viral foci or signs of phage lytic replication (white arrowheads). Experiment was independently replicated at least four times with similar results. **(c)** Maximum intensity projection images of MMC-induced *Plesiomonas* Phollow virocells. Each panel shows a representative image of fluorescent viral foci production and organization, which are similar to those in *E. coli* Phollow virocells. Magenta dashed lines mark the cell perimeter. **(d)** Alignment between P2-like prophage genomes of *Plesiomonas* (top) and *E. coli* HS (bottom). Regions of homology between the two genomes are based on percent amino acid identity (vertical gray bars), with an average genome-wide identity of 59%. The gene encoding the major capsid protein GpN, which was modified in each prophage with a Spy- or SnoopTag, is highlighted in amber. ‘Moron’ accessory regions are also indicated; ‘moron 2’ (magenta arrow) is the site where a chloramphenicol resistance gene was inserted to enable quantification of lysogen-forming units.

We next combined Phollow with optically transparent larval zebrafish, which enable direct visualization of microbial communities within a vertebrate intestine^25–27^ (Fig. 4a and Supplementary Movie 3). We colonized the intestines of germ-free zebrafish with AausFP1 *E. coli* Phollow virocells and induced lytic replication with trimethoprim (Fig. 4b). Within 4 h of administering trimethoprim, clouds of viral particles manifested throughout the gut, from the oesophagus to the distal gut (Fig. 4c,d). Outbreaks of lytic replication were acute, tapering off ~8 h post treatment, with little trace of viral particles after 24 h. Notably, most untreated animals did not harbour viral particles but in some instances, we observed spontaneous lytic replication (Fig. 4c(i) and Extended Data Fig. 8a). Trimethoprim-induced lytic replication within the gut was accompanied by the emergence of virions in the external water environment (Fig. 4e). We surmise that virions are expelled from the intestine since we did not find virions in the water in the absence of animal hosts (Extended Data Fig. 8b). We confirmed that patterns of Phollow phage outbreaks revealed by live imaging were mirrored by fluctuations in numbers of infectious virions. At 4 h post induction, the majority of samples contained significantly higher numbers of lysogen-forming units (LFUs) compared with untreated animals (Fig. 4f, left). By 24 h post treatment, LFUs returned to baseline. Within the water, LFUs generally followed the same trend, but notably, LFU abundance in treated samples was relatively sustained over the 24 h sampling period (Fig. 4f, right). These observations suggest that, following trimethoprim treatment, viral particles are expelled from the gut and persist within the water.

**Extended Data Fig. 9:**
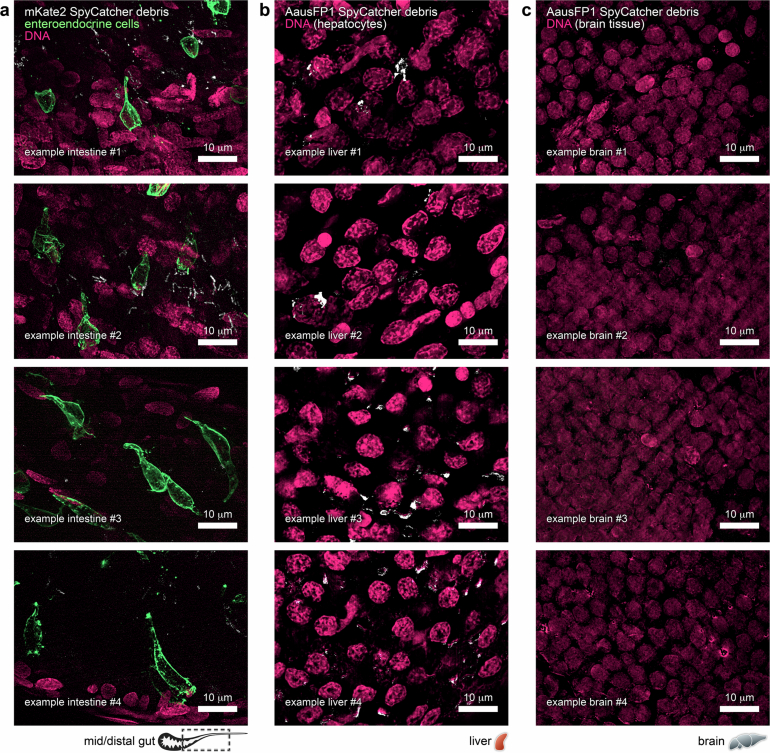
*Plesiomonas* SpyCatcher control strains do not produce viral-like particles that accumulate within EECs or extraintestinal tissues. *Plesiomonas* SpyCatcher control strains were used to colonize germ-free larval zebrafish and treated with trimethoprim as diagrammed in Fig. 4b. At 4 h post-treatment, (**a**) EECs, (**b**) livers, and (**c**) brains were imaged as in Fig. 4h–i. For each cell/tissue, four examples are shown from different animals. In (**a**) and (**c**), fluorescent SpyCatcher signal is not found within EECs or the brain, respectively. In (**b**), much of the SpyCatcher signal is from autofluorescent structures in the liver but may also reflect some translocation of SpyCatcher protein from the gut.

Our findings motivated us to test whether a similar phage from a different bacterial host exhibits distinct or overlapping behaviours within the gut. We identified a functional P2-like prophage harboured by a zebrafish gut-derived strain of *Plesiomonas*^28,29^, which we named ‘DuoZ11’ (Extended Data Fig. 8c,d). *Plesiomonas* Phollow virocells displayed patterns of intracellular virion assembly and extracellular dispersal similar to *E. coli* Phollow virocells (Extended Data Fig. 8c). Following the induction scheme in Fig. 4b, *Plesiomonas* also gave rise to clouds of virions throughout the gut (Fig. 4g and Supplementary Movie 4). However, DuoZ11 Phollow phages appeared to be quickly internalized by cells lining the gut (Supplementary Movie 5). We identified one of the intestinal cell types to be enteroendocrine cells (EECs), which we confirmed using *nkx2.2a:megfp* transgenic zebrafish^30,31^ (Fig. 4h and Supplementary Movie 6). Moreover, *Plesiomonas*-derived virions rapidly disseminated from the gut to extraintestinal sites, accumulating in the liver within lipid droplet-like compartments (Fig. 4i). Patches of viral particles (and potentially capsid debris) were also found in the vasculature, including within blood vessels of the brain (Fig. 4j). We confirmed that the fluorescent signal within EECs and extraintestinal tissues was most probably viral particles rather than non-specific SpyCatcher debris by testing *Plesiomonas* strains expressing only the SpyCatcher protein and carrying an unmodified wild-type DuoZ11 prophage. We found that induction of SpyCatcher control strains did not lead to the presence of viral-like particles in EECs, liver or brain (Extended Data Fig. 9). Finally, similar to *E. coli* virions, those from *Plesiomonas* turned over rapidly and were not detected within the gut or extraintestinal tissues at 24 h post-trimethoprim treatment. Together, these experiments, expedited by Phollow, show that P2-like phages from different bacterial hosts can share overlapping characteristics but that they also display phage or bacterial strain-dependent activities.

### Tracing interbacterial phage transmission in vitro

**Fig. 5:**
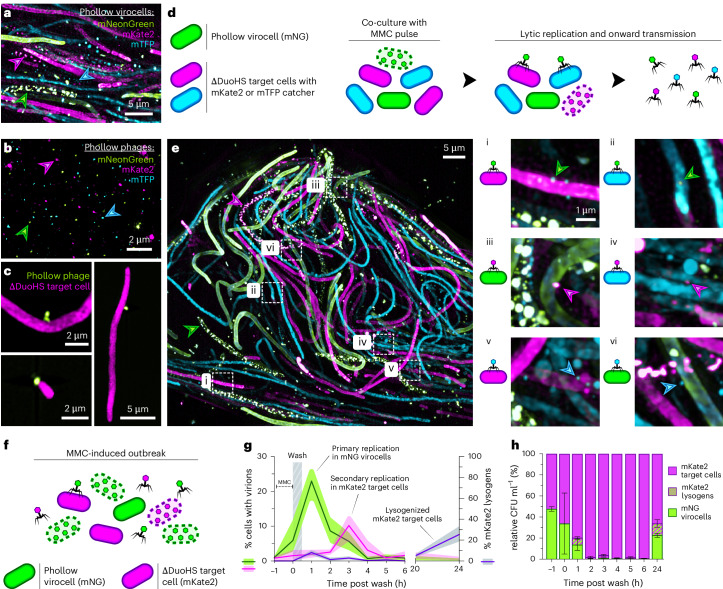
Tracing interbacterial phage transmission in vitro. **a**, Maximum intensity projection image of a mixed culture containing mNeonGreen, mKate2 and mTFP *E. coli* Phollow virocells treated with MMC. Green (mNeonGreen), magenta (mKate2) and cyan (mTFP) arrowheads indicate cells harbouring lytically replicating Phollow phage. **b**, Maximum intensity projection image of purified mNeonGreen, mKate2 and mTFP Phollow phage virions. Green (mNeonGreen), magenta (mKate2) and cyan (mTFP) arrowheads indicate each of the Phollow phage types. **c**, Maximum intensity projection images of mNeonGreen Phollow phage virions binding to mKate2 ∆DuoHS *E. coli* target cells. **d**, Diagram of three-member community (left) and MMC induction scheme (right) for imaging interbacterial phage transmission. **e**, Left: maximum intensity projection image of the three-member community diagrammed in **d** following MMC treatment. Green and magenta arrowheads indicate Phollow virocells harbouring lytically replicating phage. Right: insets showing mNG (**i**,**ii**), mKate2 (**iii**,**iv**) and mTFP (**v**,**vi**) Phollow phage landing on each bacterial community member. **f**, Diagram of two-member community for tracing interbacterial phage transmission dynamics. **g**, Left *y* axis: quantification of lytic replication in mNG virocells (green line) and mKate2 target cells (magenta line) by imaging flow cytometry. Right *y* axis: enumeration of lysogenized mKate2 target cells (purple line). Line represents the average and shaded regions indicate minimum and maximum of 3 biological replicates. **h**, Enumeration of community composition from **g** by differential plating. Data are presented as average relative abundances; bars indicate s.e.m. of 3 biological replicates.

To monitor phage transmission in vitro, we first identified three fluorescent tags that enabled simultaneous visualization of intracellular and extracellular virions (Fig. 5a–c). We then assembled a three-member community comprising mNG *E. coli* Phollow virocells and two *E. coli* target cells expressing mKate2 or mTFP SpyCatcher proteins (Fig. 5d). Inducing lytic replication within this community led to dispersal of mNG Phollow phages, which were found landing on and replicating within each target cell (Fig. 5e(i,ii)). Strikingly, mKate2 and mTFP Phollow phages were also found landing on each cell type within the community, evidence of onward transmission (Fig. 5e(iii–vi)).

We next used Phollow to quantitatively track phage transmission in a two-member community of mNG *E. coli* Phollow virocells and mKate2 *E. coli* target cells (Fig. 5f). For transmission experiments, we used Phollow phages carrying a chloramphenicol resistance gene to monitor lysogeny of target cells (see Methods). MMC induced an acute wave of mNG phage lytic replication that coincided with a sharp decline in the mNG virocell population (Fig. 5g,h). mNG phages infecting mKate2 target cells triggered a subsequent wave of mKate2 phage lytic replication, with both waves subsiding by 24 h (Fig. 5g). The overall outbreak produced shifts in community composition, with the emergence of a new mKate2 virocell population generated by lysogeny (Fig. 5g,h). This experiment demonstrates how Phollow can reveal the underlying transmission dynamics that shape bacterial communities.

### Mapping dynamics of phage replication within the gut

**Fig. 6:**
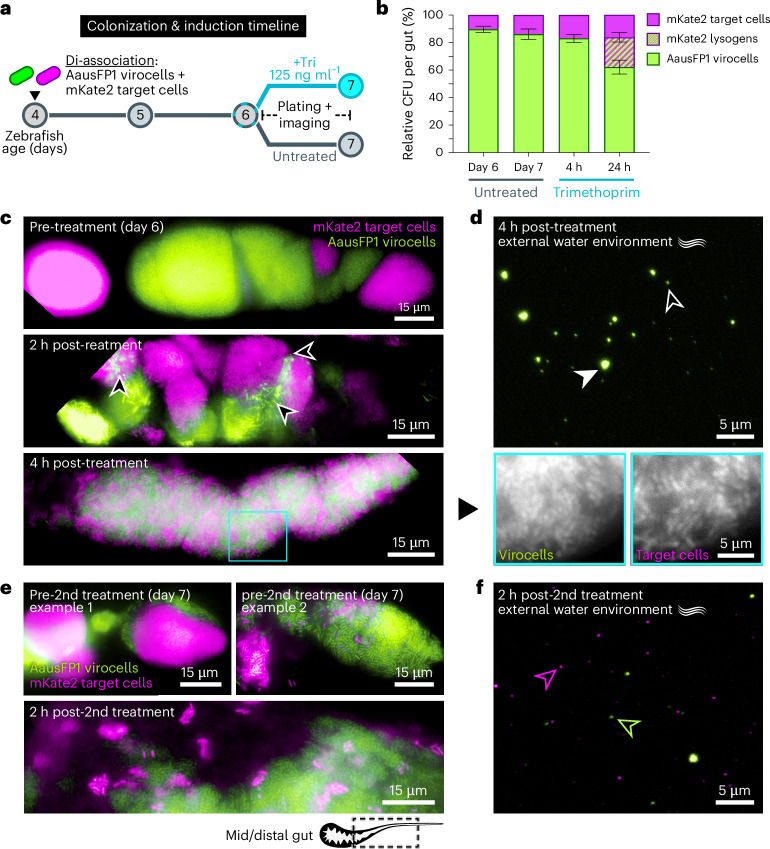
Mapping spatiotemporal dynamics of phage replication regimes within the gut. **a**, Bacterial colonization and antibiotic induction timeline. **b**, Enumeration of community composition by differential plating. Data are presented as average relative abundances; bars indicate s.e.m. of 3 biological replicates comprising 30 total fish at each timepoint and condition. **c**, Fluorescence microscopy images of AausFP1 virocell/mKate2 target cell gut communities at pre (top), 2 h post (middle) and 4 h post-trimethoprim treatment. Each image was taken from different zebrafish hosts. Black arrowheads in the middle image indicate areas containing lytically replicating phage. Due to how intestinal tissues were oriented during acquisition, images have been rotated to face anterior–posterior positions, cropped and placed on a black background, creating clipped edges in some panels. Bottom insets: AausFP1 virocell and mKate2 target cell channels shown separately to highlight degree of community mixing. **d**, Representative fluorescence microscopy image of a water sample at 4 h post-trimethoprim treatment. White arrowhead indicates a virion aggregate; black arrowhead indicates a single viral particle. **e**, Fluorescence microscopy images of AausFP1 virocell/mKate2 target cell gut communities before (top) and 2 h after (bottom) a second post-trimethoprim treatment. Each image is from different zebrafish hosts. **f**, Representative fluorescence microscopy image of a water sample 2 h after a second trimethoprim treatment. Magenta arrowhead indicates an mKate2 Phollow phage virion; green arrowhead indicates an AausFP1 Phollow phage virion.

**Extended Data Fig. 10:**
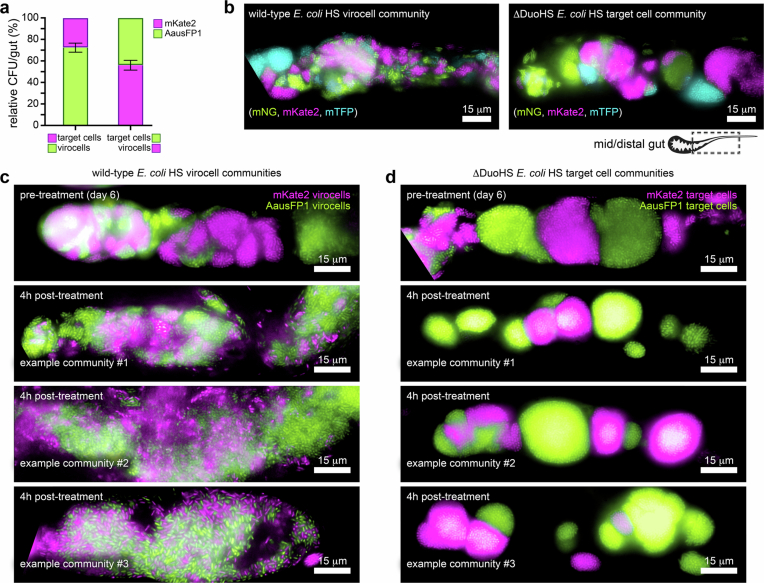
Characterization of interbacterial interactions and community spatial organization during antibiotic-induced phage outbreaks within the gut. **(a)** Determination of possible fitness tradeoffs resulting from the expression of mKate2 or AausFP1 catcher peptides. Two-member gut communities comprising wild-type *E. coli* HS virocells and ΔDuoHS *E. coli* HS target cells were assembled as diagrammed in Fig. 6a. The community represented by the plot on the left contains AausFP1 virocells and mKate2 target cells and was established and quantified as in Fig. 6b ‘Day 6, untreated’. The community represented by the plot on the right contains mKate2 virocells and AausFP1 target cells. Data are presented as average relative abundances; bars indicate standard error of the mean (SEM) from 2 biological replicates comprising 20 total fish in each condition. **(b)** Three-member communities containing differentially tagged wild-type *E. coli* HS virocells (left) or ΔDuoHS *E. coli* HS target cells (right) show the intrinsic spatial organization of bacteria within the larval zebrafish gut. Bacterial strains in each panel were engineered to constitutively express either mNG, mKate2, or mTFP. Animals were colonized with each community for two days starting on day 4-post fertilization prior to imaging. **(c,d)** Fluorescent microscopy images of two-member virocell (**c**) or target cell (**d**) gut communities prior to and 4 h post-trimethoprim treatment. A single representative image is shown for the pre-treatment time point and three example images from different zebrafish hosts are shown for the 4 h time point. Due to how intestinal tissues were oriented during acquisition, images have been rotated, cropped, and placed on a black background, creating clipped edges in some panels.

To track phage transmission dynamics within the gut, we colonized germ-free zebrafish with a two-member bacterial community composed of AausFP1 *E. coli* Phollow virocells and mKate2 *E. coli* target cells (Fig. 6a). Before induction, AausFP1 virocells displayed a colonization advantage over mKate2 target cells, which may be due in part to slight fitness trade-offs associated with expressing different SpyCatcher proteins (Fig. 6b ‘untreated’ and Extended Data Fig. 10a). Both populations showed strong spatial segregation, creating a patchwork of clonal clusters (Fig. 6c, top). This pattern resembles those previously observed in zebrafish gut communities^32^ and is produced by isogenic wild-type and ΔDuoHS *E. coli* populations (Extended Data Fig. 10b). After administering trimethoprim, lytic replication in AausFP1 virocells was detected as early as 2 h post treatment, but surprisingly, this was followed by a rapid reconfiguration of community organization (Fig. 6c, middle and bottom). By 4 h post treatment, the community displayed a striking level of spatial mixing, which we found is probably driven by the outbreak of phage lytic replication and not antibiotic treatment alone (Extended Data Fig. 10c,d). Notably, the relative abundances of each member at the 4 h timepoint were mostly unchanged, and we did not detect instances of lysogeny in mKate2 target cells (Fig. 6b). In addition, only AausFP1 virions had been expelled into the water, suggesting a lack of interbacterial transmission (Fig. 6d).

We considered the possibility that phage transmission in the gut was altered or delayed compared with the dynamics we observed in vitro. No viral particles were found within the gut at 24 h post treatment, but intriguingly, communities showed substantial recovery of a pre-treatment spatial structure (Fig. 6e, top). Probing community composition revealed the presence of mKate2 virocells (formed by lysogeny) (Fig. 6b). This observation indicates that between 4 h and 24 h, horizontal transmission of AausFP1 Phollow phages had occurred, which we confirmed by inducing a second wave of lytic replication. We again observed immediate spatial fragmentation of the bacterial community (Fig. 6e, bottom), but during the second treatment, both AausFP1 and mKate2 Phollow phages were expelled into the water (Fig. 6f). We surmise that the source of mKate2 virions was probably mKate2 virocells formed during the first wave of AausFP1 phage lytic replication. Altogether, Phollow revealed that there is a co-dynamic between the spatial organization of gut bacterial communities and phage dispersal and transmission.

## Discussion

Previous live imaging approaches have uncovered amazing details of phage biology^24,33–36^, but capturing phage replication dynamics within microbiomes and animal hosts requires additional strategies. Our solution is Phollow, which blends new genetic tools and experimental models to investigate phage lytic replication and transmission in situ (Fig. 1). We showed that Phollow phages are amenable to both live imaging and flow virometry techniques (Figs. 2 and 3), which has the potential to facilitate novel experimental schemes aimed at dissecting the epidemiology of phage outbreaks (Fig. 5). Emphasizing this potential, Phollow exposed new aspects of P2-like phage replication, namely, the formation of virion aggregates that rapidly disassemble into single particles upon bacterial lysis (Supplementary Movie 2). P2-like phage virion aggregates could be a consequence of biophysical constraints within the cytosol, but we posit that they may facilitate evasion of antiphage defence systems^37^ or shield virions from harsh extracellular environments^38,39^.

Visualizing lytic replication within the gut revealed previously unseen patterns of dispersal and biogeography (Fig. 4). Antibiotic-induced outbreaks occurred on rapid timescales and generated virion clouds that filled the luminal space (Supplementary Movie 4). However, the residency of virions within the gut was short lived. The fast turnover of virions could be driven by intestinal mechanics, which is known to purge bacterial populations and control community composition^26,32^. Equally surprising was the speed with which phage outbreaks sparked transformations in the spatial structure of bacterial communities (Fig. 6c and Extended Data Fig. 10c,d). Our observations highlight the intriguing possibility that phage replication regimes create patterns of organization by affecting bacterial growth and death across a community.

Our finding that DuoZ11 Phollow phages from *Plesiomonas* readily associate with and disseminate across zebrafish tissues provides new opportunities for studying how prokaryotic viruses interact with and modulate eukaryotic biology^40–43^ (Fig. 4h–j). In mammals, translocation of phages across the gut epithelium is suggested to be an active and ongoing process^44^. We find that EECs may be an important conduit for phage dissemination (Supplementary Movies 5 and 6). The prospect that EECs are involved in phage translocation is compelling given that they monitor luminal nutrients and microbial products, and can modulate metabolism and immunity^45^. Beyond the gut, the presence of virions within the liver (Fig. 4i) is consistent with studies showing that phages injected into the bloodstream are cleared by hepatocytes^46,47^. Moreover, in pigs and macaques, the DNA of resident gut phages can be recovered from parenchymal organs, including the liver^48^. We have not verified whether phages accumulating in extraintestinal sites are infectious. Based on studies in mice, we expect infectivity to decay over time. However, it was recently shown that the nucleic acid of some phages can have immunomodulatory activities^40,43,49,50^. Therefore, it will be interesting to investigate whether *Plesiomonas*-derived DuoZ11 phages also influence inflammatory tone, either as intact virions or through their biochemical and molecular components. Lastly, it remains unknown why DuoZ11 (from *Plesiomonas*) and not DuoHS (from *E. coli* HS) readily disseminate from the gut. These two phages share considerable homology, but each also carries unique and uncharacterized genes (Extended Data Fig. 8d). Further comparative work is therefore needed to identify the traits that underlie their potential to interact with eukaryotic cells and tissues.

The strength of Phollow is that it enables the nanoscopic lives of bacterial viruses to be directly visualized. Although we used Phollow to illuminate facets of P2-like phage biology, we expect that it can accelerate the exploration of other phage lineages. The immense diversity of phages and uncharacterized viral genetic dark matter make predicting a phage’s activity in its natural environment nearly impossible. We think that Phollow can help overcome this challenge. We note, however, that Phollow has some limitations. As with any approach involving genetic modifications, there is a chance that fluorescently marking virions disrupts their native functions. For example, phages that rely on capsid-mediated interactions may be especially sensitive to Phollow tagging^51,52^. In addition, although we did not explicitly probe the evolutionary consequences of Phollow tagging, it could impose divergent selective pressures in some contexts. Another limitation is the reliance on oxygen-requiring fluorescent proteins, potentially restricting Phollow’s use in anaerobic environments. However, we anticipate that oxygen-independent fluorescent reporters could be easily incorporated into the current Phollow tagging system^53,54^. Finally, live imaging techniques alone are limited in their ability to extract information from biological systems. We envision that it will require a combination of conventional and novel approaches, as well as new model systems, to piece together the dynamic and nested life cycles of phages within the context of complex microbiomes.

## Methods

### Animal care

All experiments with zebrafish were done in accordance with protocols approved by the University of California Irvine Institutional Animal Care and Use Committee (protocol AUP-23-126) and followed standard protocols^55^. Specific handling and housing of animals during experiments are described in detail under the section ‘Gnotobiology’. All zebrafish used in this study were larvae, between the ages of 4 and 7 days post fertilization. Sex differentiation occurs later in zebrafish development and thus was not a factor in our experiments. Zebrafish lines used in this study included wild-type AB and zebrafish carrying the TgBAC(*nkx2.2a:megfp*) transgene that expresses a membrane-tethered enhanced green fluorescent protein (mEGFP) under control of *nkx2.2a* regulatory sequences carried on a bacterial artificial chromosome (BAC)^30,31^.

### Gnotobiology

Wild-type (AB) and transgenic TgBAC(*nkx2.2a:megfp*)^30,31^ zebrafish embryos were derived germ free and colonized with bacterial strains as previously described^56^, with slight modifications. Briefly, fertilized eggs from adult mating pairs were collected and incubated in sterile embryo media (EM) containing ampicillin (100 μg ml^−1^), gentamicin (10 μg ml^−1^), amphotericin B (250 ng ml^−1^), tetracycline (1 μg ml^−1^) and chloramphenicol (1 μg ml^−1^) for ~6 h. Embryos were then washed in EM containing 0.1% polyvinylpyrrolidone-iodine followed by EM containing 0.003% sodium hypochlorite. Sterilized embryos were distributed into T25 tissue culture flasks containing 15 ml sterile EM at a density of one embryo per millilitre and incubated at 28.5 °C before bacterial colonization. Embryos were sustained on yolk-derived nutrients and were not fed during experiments. For bacterial association studies, bacterial strains were grown overnight as described in ‘Bacterial strains and culture’. Bacteria were then prepared for inoculation by pelleting 1 ml of culture for 2 min at 7,000 × *g* and washing once in sterile EM. Bacterial strains were individually added to the water of single flasks containing 4-day-old larval zebrafish at a final density of 10^6^ bacteria per ml.

### Bacterial strains and culture

All wild and recombinant bacterial strains used or created in this study are listed in Supplementary Data 1. Archived stocks of bacteria were maintained in 25% glycerol at −80 °C. Before manipulations or experiments, bacteria were directly inoculated into 5 ml TSB media (MP Biomedicals, VWR) and grown for ~16 h (overnight) with shaking at 37 °C, except for *Plesiomonas* ZOR0011, which was grown at 30 °C. For growth on solid media, tryptic soy agar (TSA; Hardy Diagnostics, VWR) was used. When specified, media were supplemented with 10 µg ml^−1^ gentamicin or 20 µg ml^−1^ chloramphenicol.

### Molecular techniques and strain construction

Plasmids were constructed using standard molecular cloning techniques as previously described^28^ and in vivo assembly (IVA)^57^, using primers listed in Supplementary Data 1. All engineering plasmids and recombinant strains were sequence and/or PCR confirmed.

#### Phollow phage and virocell engineering

SpyCatcher^17^ (Addgene 35044), SpyCatcher003 (ref. ^21^) (Addgene 133449) and SnoopCatcher^22^ (Addgene 72322) plasmids were purchased from Addgene, and IVA was used to fuse each Catcher gene to the N terminus of individual fluorescent proteins, along with a GSSGS linker. Catcher fusions were cloned into the pXS^28^ expression scaffold. Next, Catcher fusion genes along with the constitutive pTac promoter and transcriptional terminator contained within pXS were subcloned into pTn7xTS^28^ for transposon-based insertion into the *attTn7* site of the bacterial chromosome. SpyTag, SpyTag003 and SnoopTag were fused to the C terminus of the phage major capsid protein (GpN) with an SGGGSG linker in pAX1 (ref. ^28^) using IVA. The resulting plasmids were used for allelic exchange to generate a markerless modification of the gene encoding GpN within the prophage genome in the chromosome of *E. coli* HS or *Plesiomonas* ZOR0011 as previously described^28^. Further details on plasmid design and construction are provided in Supplementary Data 1.

#### Creation of ΔDuoHS ‘target cell’, Δ*tum* and *lexA*(Ind-) strains of *E. coli* HS

Mutant strains were created by markerless allelic exchange using pAX1 (ref. ^28^). For ΔDuoHS ‘target cell’ strains used as recipients for DuoHS phage transmission experiments and infectivity (LFU) assays, allelic exchange cassettes were designed to delete the entire DuoHS prophage genome while reconstituting the attachment site (*attB*) on the bacterial chromosome to enable DuoHS phage integration and lysogeny. For the Δ*tum* mutant strain, an in-frame deletion of the prophage gene encoding the Tum antirepressor was made using an allelic exchange cassette that fused the *tum* start and stop codons. For the *lexA*(Ind-) strain, the *lexA* open reading frame was engineered using allelic exchange to encode a G85D residue mutation in the LexA protein (via a GGT to GAC codon change). See Supplementary Data 1 for plasmid construction details.

#### Creation of fluorescent ‘SOS’ reporter

A fluorescent SOS reporter was engineered by fusing the promoter region upstream of the *E. coli* HS *recA* gene (locus tag ECHS_RS13985) (P*recA*) with an open reading frame encoding mNeonGreen within a pTn7xTS vector. See Supplementary Data 1 for plasmid construction details. The reporter was inserted into the *attTn7* site of the bacterial chromosome via Tn7 tagging as previously described^28^.

#### Creation of marked DuoHS phage variants for measuring infectivity by LFU assays

To measure the infectivity of DuoHS phage variants, a gene encoding resistance to chloramphenicol (from pKD3 (ref. ^58^)) was inserted into the second moron region^19^ of the DuoHS prophage (between tail genes *gpH* and *gpFI*) using the lambda-red-mediated linear transformation system^58,59^. Briefly, the chloramphenicol resistance gene from the pKD3 plasmid was PCR amplified with 40-base pair overhangs specific to the 5’ and 3’ ends of the second moron region. The resulting PCR amplicon was then introduced via electroporation into *E. coli* HS strains carrying pKM208, which encodes an isopropyl-β-d-thiogalactopyranoside-inducible lambda red recombinase. Successful recombinants were selected on plates containing chloramphenicol (creating DuoHS *moron::clm*). As a control for LFU infectivity assays, an *E. coli* HS ‘donor’ strain (HS *DuoHS::clm*) was created by lambda red recombination in which the chloramphenicol resistance gene was used to replace the entire DuoHS prophage.

### Disc diffusion tests to visualize SOS response by MMC

*E. coli* HS strains carrying the SOS reporter were grown overnight in TSB at 37 °C with shaking. The following day, cultures were diluted 1:100 in fresh TSB and spread onto agar plates using a cotton swab to create a lawn of growth. Antibiotic assay discs were placed at the centre of each plate and loaded with 5 µl of 500 ng ml^−1^ MMC. Plates were incubated overnight at 37 °C and imaged using a Leica MZ10F fluorescence stereomicroscope.

### In vitro characterization of DuoHS phage induction

#### Induction of DuoHS phage lytic replication

Induction of the DuoHS prophage was achieved by treating *E. coli* HS strains with a pulse of MMC (Goldbio). Initially, overnight bacterial cultures were diluted 1:100 in fresh TSB media and incubated at 37 °C with shaking for 1 h. MMC was then added to the media at a final concentration of 8 µg ml^−1^ for 1 h. Following this acute treatment, the cultures were centrifuged at 4,000 × *g* for 5 min. Bacterial pellets were washed twice with 0.7% saline to remove MMC, an important step to prevent interference with phage replication. Finally, bacterial pellets were suspended in fresh TSB and returned to 37 °C with shaking to monitor bacterial growth, lysis and phage induction. Note that the washing steps can cause a slight decrease in optical density due to cell loss, which is independent of bacterial lysis

#### Lysis curves

Bacterial lysis due to phage induction was evaluated by monitoring changes in culture turbidity using a FLUOstar Omega plate reader and the Omega software v.5.7. Following MMC treatment and subsequent wash steps, bacterial cultures were dispensed at 200 μl per well into a sterile 96-well clear flat-bottom tissue culture-treated microplate (Greiner Bio-One) and incubated at 37 °C with shaking. Optical density measurements at 600 nm were recorded every 30 min until stationary phase was reached. Growth measurements were repeated at least three independent times with consistent results. Data were exported and graphed using GraphPad Prism 6 software.

#### Flow cytometry

For flow cytometry, samples were collected at regular intervals spanning the initial MMC treatment to bacterial lysis. Bacterial cultures (100 μl) were centrifuged at 4,000 × *g* for 5 min. The resulting pellets were then suspended in 4% paraformaldehyde (PFA; Biotium) solution in phosphate buffer saline (PBS; pH 7.4). Samples were fixed in this solution for 30 min at room temperature, and then pelleted and suspended in 100 μl of sterile 0.7% saline. Samples were stored at 4 °C until further analysis.

To measure SOS reporter activity, samples were analysed on a Novocyte flow cytometer (Agilent) equipped with NovoExpress software (v.1.5.6). A total of 10,000 events were collected. Cells were first identified based on their forward and side scatter profile and subsequently analysed for mNG signal. Gates to identify SOS positive events were calibrated using an untagged wild-type *E. coli* HS strain as a negative control and an *E. coli* HS strain constitutively expressing mNG as a positive control. Data were analysed using FlowJo 10.10 software (Treestar).

Imaging flow cytometry was performed using an ImageStreamX MKII cytometer (Cytek Biosciences) using ×60 magnification. Brightfield images of the bacterial cells were captured using channels 1 and 7. The 488 nm laser was used in channel 2 to visualize mNeonGreen fluorescence and, when necessary, the 561 nm laser was used in channel 4 to capture mKate2 fluorescence. All other channels were disabled during data acquisition. A total of 10,000 events per sample were collected using the INSPIRE ISX software v.200.1.681.0 software. The raw image files were saved and subsequently analysed using a custom data analysis template generated in IDEAS software v.6.2. Samples were gated based on pixel intensity variance and contrast morphology features generated with the machine learning algorithm included in IDEAS software (Supplementary Data 2). Gates were manually curated and adjusted by visually inspecting and excluding inappropriate events. Data from three independent experiments were exported and graphed using GraphPad Prism 6 software.

#### Live imaging

To prepare for imaging, 1 μl of either bacterial or phage samples was placed onto glass slides and covered with a glass coverslip. Routine fluorescence microscopy was performed using an Echo Revolve microscope with a ×60 oil immersion objective. For super-resolution microscopy, bacterial cultures were embedded on 2.5% low-melting-point agarose pads and covered with #15H high-performance coverslips (Thorlabs).

For the staining of bacterial or phage DNA, 1 µl of the sample was combined with 100 µg ml^−1^ Hoechst 33342 dye (ImmunoChemistry Technologies) before mounting the samples onto a microscope slide. To selectively stain extracellular DNA, bacterial cultures were treated with the membrane impermeable DNA dye ethidium homodimer-III (EthD-III; Biotium) following manufacturer instructions. For membrane visualization, samples were stained with 10 µM FM4-64 (Biotium) before the mounting procedure.

#### Time-lapse imaging

To capture time-lapse images of Phollow phage induction, bacteria were first grown overnight in 5 ml TSB at 37 °C with shaking. The next day, cultures were diluted 1:100 in fresh TSB and grown for an additional hour under identical conditions. Then, 120 μl of the bacterial culture was transferred into a well of an 8-well #1.5 high-precision slide chamber (Cellvis). To improve immobilization of bacterial cells, the chamber was previously coated with 0.1% poly-d-lysine solution (Thermo Scientific). To further create a firmly attached thin film of bacterial cells, the chamber was centrifuged at 1,400 × *g* for 15 min at room temperature. Post centrifugation, the supernatant was aspirated and non-adherent cells were gently removed by washing three times with 200 μl of a 0.7% saline solution. Finally, 150 μl of TSB supplemented with 0.2% low-melt agarose (Sigma) and 8 μg ml^−1^ MMC was added to the well. The chamber was left at room temperature for 5 min to ensure complete solidification of the agar before mounting the sample on the Zeiss Elyra 7 microscope. Imaging took place at 10-min intervals using structured illumination.

### Super-resolution microscopy

Imaging was performed using a Zeiss Elyra 7 microscope equipped with an alpha Plan-Apochromat ×63/1.46 oil immersion lens. Imaging was performed using Zeiss Zen Black v.3.2 software. Unless otherwise specified, total internal reflection fluorescence with ultra-high-performance illumination was employed. The images were captured using laser lines at 405 nm for Hoechst 33342 dye, CF405-phalloidin and mTFP; 488 nm for AausFP1, GFP and mNG; 561 nm for JF549 dye^60^ and mKate2; and 660 nm for BDP lipid stain. A bandpass emission filter was centred at 560 nm in camera 2. The exposure time was consistently set to 25 ms, while the excitation laser power was maintained at 250 mW for all bacterial samples and 500 mW for zebrafish whole mounts. *Z*-stack images were acquired with the optimal slicing mode at a 0.085-µm step interval for single-point bacterial samples, leap mode at 0.125-μm intervals for bacterial time lapses and leap mode at 0.256 μm for all zebrafish samples. For data reconstruction, Lattice 3D-SIM^2^ integrated into ZEN Black 2.3 software was utilized, scaling images to the raw data.

Super-resolution images of a whole zebrafish mount were taken with an alpha Plan-Apochromat ×40 water immersion objective on a Zeiss LSM 980 Airyscan 2.0 microscope using the Airyscan SR mode. Imaging was performed using Zeiss Zen Blue v.3.2 software. For the acquisition of a multichannel *z*-stack image, the 405 nm, 488 nm and 561 nm lasers were used to image actin stained with CF405-phalloidin (Biotium), AausFP1 virocells and DNA stained with JF549 dye^60^ (Janelia Materials), respectively. The images were processed with Airyscan Deconvolution algorithm.

### Image processing and analysis

Maximum intensity projections, photobleaching corrections^61^ and *z*-depth colouring schemes were processed using FIJI (ImageJ) 2.14/1.54f software^62^. Pseudo-coloured images were processed to consistently represent 1.36 μm of total sample depth (16 images captured using 0.085-μm steps). Three-dimensional renderings were generated with Imaris 10.1.1.

Manual creation of channel masks was necessary for images in Fig. 4a,c(iii),d,g, to account for the differences in fluorescence intensity between bacteria and phage. Custom lookup tables were also used to enable better visualization of phage virions (Supplementary Data 3). In addition, masks were created in Fig. 4a to reduce spectral overlap and emphasize the unique signal of each fluorescence channel.

Analysis of intracellular viral foci was done using built-in thresholding, particle analysis and area measurement tools in Fiji^62^. Cell lengths and number of viral foci per cell were quantified using maximum intensity projection images acquired by super-resolution microscopy. Surface area (2D) measurements of viral foci were done using images of 3D renderings generated in Imaris and acquired by electron microscopy. A single DuoHS capsid (50–60 nm) produces a 2D log_10_(surface area) of 3.29–3.45 nm^2^.

### Expansion microscopy

Our ExM methods were based on published protocols^63–66^ and optimized to achieve a 25× expansion for phage-only samples and a 10× expansion for samples containing bacterial cells.

#### Sample preparation

To prepare for ExM, *E. coli* HS and *E. coli* HS TTW210 (which encodes a ‘dark’ Phollow phage) were treated with MMC as described above to induce phage lytic replication. Samples were collected 1 h after the MMC pulse and centrifuged at 10,000 × *g* for 1 min to separate bacteria in the pellet from virions in the supernatant. The phage-containing supernatants were further purified using a 10% chloroform solution.

#### Fixation

Various fixation protocols compatible with ExM yield different structural preservation outcomes. For bacterial cells, we achieved optimal preservation by fixing with a 4% PFA solution for 30 min, followed by three PBS washes. The samples were then permeabilized with 1% Triton X-100 (Sigma) in PBS and rinsed three additional times with PBS. For phage samples, fixation was omitted; instead they were directly incubated in the anchoring solution.

#### Anchoring

Bacterial samples were incubated in a 100 μg ml^−1^ acryloyl-X SE (Thermo Scientific) anchoring solution overnight at 4 °C. Phage samples were anchored in a solution of 0.7% formaldehyde (Sigma) and 1% acrylamide (AA; Fisher Scientific), also incubated overnight at 4 °C. All samples were mounted in 12 × 12 mm poly-d-lysine-coated coverslips before proceeding to the gelation step.

#### Gelation

The gelation process was similar for both sample types, differing only in the monomer solution composition. For bacterial samples, the monomer solution consisted of 1.1 M sodium acrylate (SA; VWR), 2.0 M AA, 50 ppm *N*,*N*’-methylenebisacrylamide (BIS; Fisher Scientific), PBS (1×), 0.5% ammonium persulfate (APS; Fisher Scientific) and 0.5% tetramethylethylenediamine (TEMED; Fisher scientific). For phage samples, the monomer solution was 10% AA, 19% SA, 0.1% *N*,*N*′-(1,2-dihydroxyethylene)bisacrylamide (DHEBA; Sigma), 0.5% APS and 0.5% TEMED. High concentrations of APS and TEMED enabled rapid polymerization, ensuring structural preservation while necessitating quick sample handling.

To initiate the gelation procedure, the lid of a 96-well plate served as a custom gelation chamber. With the lid facing up, the chamber was assembled on ice by placing a water-soaked paper towel within the lid, followed by a sheet of parafilm. A 35-μl drop of monomer solution was pipetted onto the parafilm, and a coverslip with the sample facing the solution was placed over the drop. Samples were incubated on ice for 5 min before transferring the gelation chamber to a 37 °C incubator for 2 h.

#### Denaturation and digestion

Following gelation, coverslips were gently removed and transferred to a 6-well plate containing 2 ml of denaturation buffer until the gel detached. For bacterial samples (10× expansion), the buffer was 200 mM sodium dodecyl sulfate (Genesee Scientific), 200 mM NaCl (Neta Scientific) and 50 mM Tris base (pH 9; GoldBio). For phage samples (25× expansion), the buffer was adjusted to pH 6.8. All gels were then transferred to 1.5 ml tubes with fresh denaturation buffer and incubated for 60 min at 85 °C.

Bacterial samples were subsequently washed with PBS, suspended in digestion buffer (50 mM Tris-HCl pH 8, 1 mM EDTA, 0.5% Triton X-100) containing 8 U ml^−1^ proteinase K (New England Biolabs) and 2 mg ml^−1^ lysozyme (Goldbio), and incubated for 2 h at 37 °C.

#### First expansion

All gels were transferred to 10 cm Petri dishes containing deionized (DI) water and incubated for 30 min. The water was then replaced and the incubation process repeated twice more, for a total of three cycles. Following the third incubation, the gels were placed in fresh DI water and incubated at 4 °C overnight.

#### Iterative expansion for phage samples

Initially, the expanded gels were cut into four pieces and placed in a 6-well plate on ice. Each gel piece was incubated on ice with activated neutral gel (10% AA, 0.05% DHEBA, 0.1% APS/TEMED) for three consecutive 10-min intervals. After incubation, each gel piece was placed on a microscope slide, and any excess monomer solution was gently blotted away using Kimwipes. A 22 × 22 mm coverslip was then applied, and the gel was incubated in a gelation chamber at 37 °C for 1 h.

The gels were then transferred to a 6-well plate and washed three times for 10 min on ice, using the second expansion monomer solution (14.5% AA, 10.5% SA, 0.01% BIS, 0.05% APS, 0.05% TEMED). Once again, excess monomer solution was removed with Kimwipes, and the gels were covered with a 22 × 22 mm coverslip and incubated in a gelation chamber at 37 °C for 1 h.

After the final polymerization step, the entire gel was incubated in 200 mM NaOH solution for 1 h with shaking at room temperature. This allowed the dissolution of the first and neutral gels. Following this, the gel was washed with PBS in 20-min intervals until the pH reached 7.

#### Gel staining and final expansion

Gels were equilibrated in PBS before staining. Samples from ‘dark’ Phollow strains were incubated overnight at 4 °C in 1 ml of filtered, sterilized bacterial lysate expressing Spycatcher mNG. Gels were washed three times with PBS–0.1% Tween 20 (Sigma) and resuspended in PBS.

For NHS ester staining, gels were incubated in 20 µg ml^−1^ CF405S NHS ester dye (Biotium) at 4 °C overnight, then washed four times in PBS at 10-min intervals. Finally, gels were re-expanded following the first expansion steps.

#### Microscopy

Gels were mounted on poly-d-lysine-coated glass-bottom dishes, and excess water was gently blotted with a Kimwipe. To prevent gel dehydration during imaging, 5 µl of DI water was added to the gel every hour. Imaging was conducted on a Zeiss Elyra 7 microscope with an alpha Plan-Apochromat ×63/1.46 oil immersion lens using ×1.6 and ×1 lenses. Images were acquired using a 405 nm laser line, with a consistent exposure time of 25 ms and an excitation laser power of 500 mW.

### Transmission electron microscopy

To prepare for TEM, bacteria were treated with a pulse of MMC as described above. Samples were collected 1 h after the MMC pulse and applied to 300- or 400-mesh carbon-coated copper grids (Gilder, Ted Pella) and negatively stained with 0.75% uranyl formate using established protocols^67^ and as described below. Sample concentrations were adjusted on the basis of the results from screening. Grids were negatively glow-discharged with a PELCO easiGlow (Ted Pella) to render them hydrophilic before staining. Sample (3 μl) was applied to each grid for 2 s or up to 60 s depending on sample concentration. Then, excess liquid was blotted, the grid was washed with two drops of Milli-Q water, stained with 2 drops of uranyl formate and allowed to air dry. For some preparations, the Milli-Q water wash step was omitted and replaced with stain. All samples were screened and data collected using a JEOL JEM 2100F or a JEOL JEM 2800 transmission electron microscope equipped with a Gatan OneView Microscopy Suite v.3.2.1461.0 with a (4k × 4k) camera. Resulting micrographs were analysed and contrast enhanced for clarity using FIJI^62^. This experiment was independently replicated at least twice with similar results.

### Phage purification for flow virometry

Phage lytic replication was induced as described above, and 1 ml of bacterial culture was collected 1.5–2 h post wash. Chloroform (0.1× culture volume) was added and the sample was vortexed for 1 min and incubated at room temperature for 5 min to lyse bacterial cells. The sample was centrifuged at 10,000 × *g* for 5 min to pellet bacterial cell debris, and the supernatant was moved to a new tube. DNAse I buffer (10×) was added to 1× final concentration along with 1 μl of DNAse I, and the sample was incubated at 37 °C for 30 min. EDTA (20 mM final concentration) was added to inactivate the DNAse I. NaCl (final concentration 1 M) was added and the sample was incubated on ice for 30 min and centrifuged at 10,000 × *g* for 5 min to pellet debris. The supernatant was used for flow virometry.

### Flow virometry

Flow virometry was performed on Cytek’s Northern Lights 3-laser spectral flow cytometer or Cytek’s Aurora 5-laser spectral flow cytometer using the SpectroFlow software. Phage particle-sized events were first identified and detected on an FSC-H vs SSC-H plot using Spherotech’s 0.13 μm yellow sizing beads (NFPPS-0152-5) while adjusting voltage gains for FSC and SSC. A total of 10,000–50,000 events were captured depending on the phage availability in the samples. Raw FCS files were unmixed with reference controls of each respective Phollow phage sample containing the SpyTag and SpyCatcher, and the unstained reference control was used as a prophage deletion mutant strain.

Unmixed FCS files were analysed using FCS Express v.7.22.0031. Phages were gated on the basis of their size relative to the 100 μm sizing beads described above. Single particle gating on SSC-A vs SSC-H was used to discriminate against doublets. Positive MFI gates were drawn for each respective fluorescent Catcher peptide using samples negative for the SpyTag but containing the fluorescent Catcher peptide. Data were graphed and analysed using GraphPad Prism 6 software.

### MIC determination and evaluation of phage induction with different antibiotics

To evaluate the MIC of MMC, trimethoprim (GoldBio) and ciprofloxacin (GoldBio), Phollow virocells were first grown overnight in 5 ml TSB at 37 °C with shaking. The next day, cultures were diluted 1:10^6^ in fresh TSB and grown for an additional 3 h under identical conditions. Then the bacterial cultures were diluted 1:3 into fresh TSB containing a final antibiotic concentration ranging from 31.25 to 2,000 ng ml^−1^ for MMC, 19.5 to 1,250 ng ml^−1^ for trimethoprim and 48–3,125 ng ml^−1^ for ciprofloxacin. The different cultures were dispensed into a sterile 96-well clear-bottom microplate, and optical density was measured as described above. MIC was determined as the lowest concentration required to significantly impair bacterial growth without complete eradication. To evaluate the phage induction potential of the three antibiotics, the bacteria were cultured as described for the MIC and exposed to a range of antibiotic concentrations relative to the MIC, ranging from 0.05× to 2× the MIC. Bacterial culture (130 μl) was collected 24 h after antibiotic exposure, and phages were purified from bacterial debris as described for virometry. To visually quantify phage induction at different antibiotic concentrations, 1 μl of purified phage samples was placed onto glass slides and covered with a glass coverslip. Fluorescence microscopy was performed using an Echo Revolve microscope with the Echo Pro software (v.6.4.2) and a ×60 oil immersion objective. The free virions per field of view were quantified using the particle analysis function of FIJI (ImageJ)^62^. Peak virion output for each antibiotic was quantified by flow virometry as described above.

### Infectivity assay using lysogen-forming units

Phage lytic replication was induced as described above, and 500 μl of bacterial culture was collected and centrifuged at 10,000 × *g* for 5 min to pellet bacterial cells. The supernatant was moved to a new tube, 0.1× volume of chloroform was added, and the sample was vortexed for 1 min and incubated at room temperature for 5 min before centrifuging at 10,000 × *g* for 5 min. The supernatant contained purified phage used for the infectivity assay. The recipient ‘target’ strain (*E. coli* HS ΔP2) was diluted 1:50 in 5 ml of fresh TSB supplemented with 0.5 mM CaCl_2_ and incubated at 37 °C with shaking for 30 min before infection. Purified phage (100 μl) was added to the recipient strain and incubated at 37 °C with shaking for 1 h. Dilutions of the infection culture were plated on TSA supplemented with chloramphenicol and gentamicin to select for lysogens. Plates were incubated overnight at 37 °C and colonies were counted. An *E. coli* HS *DuoHS::clm* strain was used as a donor control to account for potential phage-independent transfer of chloramphenicol resistance during LFU assays.

### In vitro phage transmission assays

To track the horizontal transmission of Phollow phages, we used a defined two-member bacterial community consisting of a phage-donor strain and a phage-naïve target strain. The donor strain, *E. coli* HS TTW255 (HS *gpN:SpyTag_Original*; *DuoHS moron::clm:* *attTn7::sco:mNG*), is an mNG Phollow virocell that harbours a chloramphenicol resistance cassette in the second moron region of the DuoHS prophage. This feature facilitates the use of selective media to monitor lysogenic conversion of the target strain. The target strain, *E. coli* HS TTW269 (ΔDuoHS; a*ttTn7::sco:mKate2*), has been genetically cured of the DuoHS prophage genome while reconstituting the *attB* attachment site to enable de novo lysogenization. In addition, this strain constitutively expresses a SpyCatcher–mKate2 fusion protein, capable of tagging newly produced ‘dark’ Phollow phage from lytic infection events.

Before the experiment, each strain was cultured independently overnight in 5 ml of TSB at 37 °C with shaking. The next day, 500 μl of the saturated cultures were mixed in a microcentrifuge tube and homogenized by vortexing for 30 s. The resulting mixture was diluted 1:100 with fresh TSB and grown for 1 h at 37 °C with shaking. Subsequently, this co-culture was treated with an MMC pulse, as described above. Following the wash steps and resuspension of the pellet in TSB, the culture was further incubated at 37 °C with agitation for 24 h. Samples of 200 μl were taken at defined intervals: before MMC addition, immediately before the washing procedure, hourly for the subsequent 6 h post wash and finally, 24 h after the washing step. At each timepoint, 100 μl of the samples were treated according to the previously described imaging flow cytometry protocol. Fixed samples were stored at 4 °C until further analysis.

The remaining sample volume was subjected to a 10-fold serial dilution using 0.7% saline solution. Aliquots of 4 µl from each dilution (ranging from undiluted to 10^−7^) were spot plated onto TSA for total viable counts and onto TSA supplemented with 20 µg ml^−1^ chloramphenicol to distinguish lysogenic fractions. After an overnight incubation at 37 °C, colony-forming units (CFUs) were enumerated. Differentiation between donor and target parental populations was achieved using a fluorescence stereomicroscope. For samples that did not yield countable colonies at the lowest dilution, a value of 1 was assigned to represent the limit of detection (LOD). Data from three independent experiments were graphed and analysed using GraphPad Prism 6 software.

### In vivo characterization of phage outbreaks in the zebrafish gut

#### Induction of phage lytic replication

Germ-free zebrafish larvae were colonized with either *E. coli* HS or *Plesiomonas* AausFP1 Phollow virocells for a duration of 48 h. AausFP1 was used for in vivo imaging experiments because it is one of the brightest known green fluorescent proteins. For co-colonization experiments, a 1:1 mix of *E. coli* HS AausFP1 donor and ΔDuoHS mKate2 cells was used for inoculation.

To induce phage lytic replication and subsequent phage dispersal, trimethoprim was added directly into flask water containing animals at a final concentration of 125 ng ml^−1^. We chose trimethoprim because it induces lytic replication over a relatively broad concentration range and probably has fewer adverse effects on animal physiology compared with MMC. Fish and flask water were sampled at 4 and 24 h post treatment to evaluate bacterial abundances, virion infectivity and to perform live imaging.

#### Collecting bacterial and viral contents from the gut

Dissection of larval zebrafish guts was done as previously described with slight modifications^26,56^. Briefly, dissected guts of tricaine-euthanized zebrafish were collected and placed in a 1.6 ml tube containing 500 μl sterile 0.7% saline and 100 μl 0.5 mm zirconium oxide beads (Next Advance). Guts were homogenized using a bullet blender tissue homogenizer (Next Advance) for 60 s on power 4.

#### Culture-based quantification of bacterial abundances

Gut and water contents were serially plated onto TSA for viable cells, or when indicated, TSA supplemented with 20 µg ml^−1^ chloramphenicol to distinguish lysogens. Plates were incubated overnight at 37 °C before enumeration of CFUs and determination of bacterial abundances. Abundance data presented throughout the main text were pooled from a minimum of 3 independent experiments (*n* = 30 dissected guts and 6 waters per condition). For samples that did not yield countable colonies at the lowest dilution, a value of 1 was assigned to represent the LOD. Data were plotted and analysed using GraphPad Prism 6 software.

#### Assessment of virion infectivity

Immediately after collecting gut and water contents, samples were treated with 10% chloroform. These mixtures were vigorously homogenized for 30 s using a vortex. Subsequently, the solutions were incubated at room temperature for 10 min before centrifugation at 10,000 × *g* for 3 min. The aqueous solutions containing purified virions were carefully extracted and further used in infectivity assays.

To infect an *E. coli* ΔDuoHS mKate2 target strain, an overnight culture of the strain was diluted 1:100 in fresh TSB and incubated at 37 °C with shaking for 30 min. The culture was then dispensed in 100 µl aliquots into the wells of a deep-well 96-well plate (Fisher Scientific). Centrifugation of the plate was performed at 4,000 × *g* for 5 min to pellet the cells. The pellets were washed twice with 1 volume of 0.7% saline solution.

Finally, the cell pellets were suspended in 100 µl of the chloroform-treated solutions. The suspensions were incubated at 37 °C with agitation for 1 h. Following the incubation, the cultures were serially diluted and plated onto TSA supplemented with chloramphenicol to select for de novo lysogens. The plates were incubated overnight at 37 °C, after which the LFU were enumerated. For samples that did not yield countable colonies at the lowest dilution, a value of 1 was assigned to represent the LOD. Data from 3 independent experiments were graphed and analysed using GraphPad Prism 6 software. Statistical analysis was performed using Kruskal–Wallis and Dunn’s multiple comparisons test.

#### Microscopy

Zebrafish larvae were fixed in 4% PFA for 2 h, after which they were washed twice with PBS. Subsequently, up to 5 larvae were placed into a microcentrifuge tube and resuspended in 200 µl of PBS containing 1 µM JF549 dye^60^ for nuclear DNA staining and 1 unit of CF405-phalloidin for actin filament staining. For experiments involving TgBAC(*nkx2.2a:megfp)* reporter fish or those displaying two-member bacterial communities, Hoechst 33342 DNA dye was utilized instead at a concentration of 2 µg ml^−1^. The larvae remained in the staining solution overnight or until ready for imaging. When indicated, stained animals were incubated with 0.5 µg ml^−1^ 650/665 BDP (Luminiprobe) overnight to stain for lipid droplets. To prepare for imaging, each specimen was positioned onto a microscope slide, ensuring the removal of all excess liquid, followed by the application of 5 µl of EverBrite mounting medium (Biotium). For dissected organs, 3% methylcellulose was used as mounting medium instead. Finally, a coverslip was placed over the larvae. Any residual mounting medium was carefully dried out, and the coverslip was securely sealed with nail polish.

For live imaging experiments, larvae were anaesthetized with 0.4% tricaine to minimize movement and then embedded in 1% low-melting-point agarose placed in 35-mm glass-bottom Petri dishes. The plate was finally filled with sterile EM.

For the super-resolution imaging of a whole animal, fixed and stained larvae were embedded in 1% low-melting-point agarose placed in 35-mm glass-bottom Petri dishes. After agarose solidification, 3 ml of deionized water was added to the plate to reduce evaporation.

Images were acquired as described in the ‘Super-resolution microscopy’ section.

### Analysis of P2-like prophage relatedness

The P2-like prophage genomes of DuoHS (from *E. coli* HS) and DuoZ11 (from *Plesiomonas* ZOR0011) were annotated with Pharokka (v.1.7.1)^68^. Default settings were applied except for flag -g ‘prodigal’, which was used as a gene caller^69^. The average amino acid identity was determined as done in ref. ^70^. For visualization, pyGenomeViz (v.1.4.1) was used with MMSeq to compare the homology of the coding sequence^71^. Gene annotation was additionally manually curated during visualization.

### Reporting summary

Further information on research design is available in the Nature Portfolio Reporting Summary linked to this article.

## Supplementary information

Supplementary Information A guide to implementing Phollow.

Reporting Summary

Supplementary Video 1 **Examples of intracellular Phollow phage organization**. 360° view of images presented in Fig. 2e and Extended Data Fig. 4, highlighting patterns of intracellular virion organization. Virions show two prevalent patterns of organization: a ‘scattered’ distribution and a ‘serpentine’ distribution. Images are pseudo-coloured according to *z*-depth, representing a total of 1.36 μm.

Supplementary Video 2 **Time-lapse movie depicting Phollow phage induction, assembly and dispersal**. Time-series images of *E. coli* HS Phollow virocells growing on agar pads containing MMC. Optical frames were generated from four tile-scanned and merged fields of view, acquired at regular 10-min intervals over a period of 370 min. The time series starts at 0 min, where individual bacterial cells appear as small white rods. As time progresses, these cells undergo filamentation, a hallmark morphological change indicative of the SOS response induced by MMC genotoxicity. Subsequently, a subpopulation of bacterial cells displays the formation of intracellular fluorescent viral foci followed by explosive cell lysis. Upon lysis, Phollow phage virions can be seen dispersing throughout the extracellular milieu.

Supplementary Video 3 **Three-dimensional scan through the body of a 7-day old larval zebrafish colonized by** ***E. coli*** **HS Phollow virocells**. Optical frames were generated from 297 tile-scanned and merged images acquired by super-resolution microscopy. DNA (magenta, JF549 dye) and actin (purple, CF405-phalloidin) highlight zebrafish anatomy and tissues, bacteria (green) localize as aggregates in the midgut.

Supplementary Video 4 **Spatial distribution of Phollow phage virions within the zebrafish gut**. Three-dimensional scan through the anterior region of a 6-day old larval zebrafish intestine colonized by AausFP1 *Plesiomonas* Phollow virocells. The video indicates the presence of bacteria and viral clouds throughout the luminal space. Images were acquired at 4 h post-trimethoprim treatment, during an outbreak of phage lytic replication. DNA (magenta, JF549 dye) and actin (purple, CF405-phalloidin) highlight the intestinal structure.

Supplementary Video 5 **Phollow phage virions are dynamic and closely associate with the intestinal mucosa**. Live imaging of the anterior region of a 6-day-old larval zebrafish intestine colonized by AausFP1 *Plesiomonas* Phollow virocells. The video shows the close association with and possible translocation of virions across the gut epithelium.

Supplementary Video 6 **Example of internalization of Phollow phage virions by enteroendocrine cells**. Three-dimensional reconstruction of intestinal tissue highlighting the co-localization of viral particles (greyscale) within an enteroendocrine cell (EEC, green). The membranes of EECs are marked using TgBAC(*nkx2.2a:megfp*) reporter fish and DNA is stained with Hoechst 33342 dye (magenta). Surface rendering and 360° view were generated from *z*-stack images using Imaris.

Supplementary Data 1 Information regarding strains and plasmid construction.

Supplementary Data 2 Imaging flow cytometry gating algorithm.

Supplementary Data 3 ImageJ lookup tables.

Supplementary Data 4 Numerical raw data used to generate figures.

## Data Availability

All numerical data underlying plots shown in main and extended data figures are provided in Supplementary Data 4. Image source data files are deposited in figshare at 10.6084/m9.figshare.28477262 (ref. ^72^).
