# Supplementary material for: Phollow reveals in situ phage transmission dynamics in the zebrafish gut microbiome at single-virion resolution
Source: Nat Microbiol. 2025 Apr 18;10(5):1067–83. doi: 10.1038/s41564-025-01981-1 (PMC12055606; doi:10.1038/s41564-025-01981-1)
Supplement: Supplementary file 1 — A guide to implementing Phollow. [file 41564_2025_1981_MOESM1_ESM.pdf]

nature microbiology

Supplementary information https://doi.org/10.1038/s41564-025-01981-1

Phollow reveals in situ phage transmission dynamics in the zebrafish gut microbiome at single-virion resolution

In the format provided by the authors and unedited

Phollow reveals in situ phage transmission dynamics in the zebrafish gut microbiome at single-virion resolution

Lizett Ortiz de Ora, Elizabeth T Wiles, Mirjam Zünd, Maria S Bañuelos, Nancy Haro-Ramirez, Diana S Suder, Naveena Ujagar, Julio Ayala Angulo, Calvin Trinh, Courtney Knitter, Shane Gonen, Dequina A Nicholas, and Travis J Wiles

A Guide to Implementing Phollow

This guide outlines how to begin implementing Phollow for live imaging-based studies of phage biology. The experimental schemes that can be facilitated by Phollow are potentially numerous and diverse. Therefore, this guide is meant as a starting point, and covers general steps and considerations for Phollow phage construction and study.

Model Phage Selection

In our study, we chose P2-like temperate phages to develop and characterize Phollow. We expect that many types of phages can be engineered and studied using the Phollow system. However, the feasibility and success of constructing new Phollow phages will depend on the availability of information about the virion structure and mechanisms of replication for each specific phage. For example, it is useful if genes encoding structural components of the virion are known (or at least can be predicted). Moreover, knowledge of the phage's replication cycle and kinetics of virion production will greatly aid characterization. It is also important that there are established procedures for genetically manipulating both phage and bacterial host genomes. Lastly, genetically modifying any organism has the potential to compromise native functions; therefore, assays for characterizing virion infectivity and interactions with host bacterial cells are necessary for validating and interpreting experimental observations.

Phollow Phage Construction (Fig. 1)

Virion protein tagging

Virions are nanoscopic, self-assembling structures with intricate geometries. To successfully tag virions while preserving their infectivity, there are at least three important considerations for determining which virion protein to modify with a Spy- or SnoopTag:

1. *Protein location, configuration, and processing*. Because Phollow tagging is based on decorating virions with fluorescent Catcher peptides, viral proteins modified with a Spy or SnoopTag will ideally be located at or near the virion surface with the tag exposed. In addition, some virion structural proteins undergo proteolytic processing. For example, the

N-terminus of the major capsid protein GpN of P2-like phages is cleaved during capsid maturation. Therefore, Spy or SnoopTags must be engineered into protein domains that are retained within the fully assembled and infectious particle.

2. *Protein function*. It is important to consider how modifying a certain virion protein with a Spy or SnoopTag might potentially alter its function. For example, tags and fluorescent Catcher peptides could disrupt normal capsid assembly and integrity or receptor binding motifs. Therefore, rationally designing and optimizing the placement of tags will help ensure successful Phollow phage construction.

3. *Protein Copy number*. Observing fluorescently marked phage by microscopy requires sufficient quantities of fluorescent Catcher peptides to bind and label virions. Viral proteins that are present at high copy numbers within mature virions will therefore make it easier to detect phages by live imaging. For example, the major capsid protein GpN is the most abundant protein within the P2 virion (~400 copies/virion). Therefore, the copy number of tagged proteins will have a significant impact on the intensity of virion labeling.

Fluorescent protein selection

Fluorescent proteins display a range of different properties such as maturation time, excitation/emission, brightness, and photostability. Because virions are small and may ultimately become sparsely labeled by fluorescent Catcher peptides, bright and photostable fluorescent proteins perform the best for live imaging of Phollow phages. Moreover, the excitation/emission characteristics of a fluorescent protein can be a critical factor when imaging multiple fluorescent reporters and under conditions with high levels of autofluorescence or low optical clarity (such as within animal tissues). In our experience, mNeonGreen performs overall the best in a variety of conditions with AausFP1 being superior for imaging virions within zebrafish tissues because of its exceptional brightness.

Fig 1. Phollow phage construction

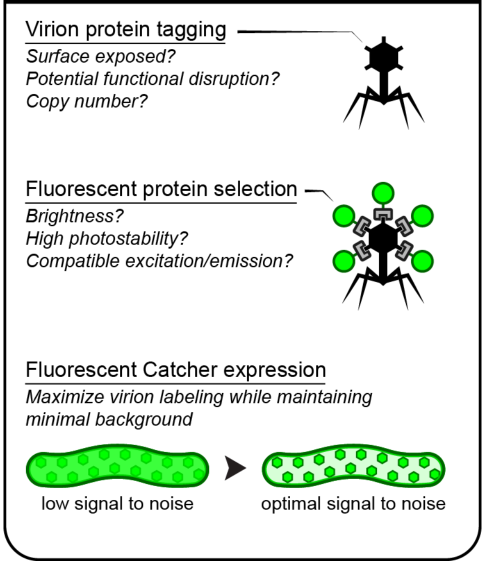

Optimizing fluorescent Catcher expression

Within Phollow virocells, fluorescent Catcher peptides are constitutively expressed and are initially spatially dispersed throughout the cell. Upon induction of phage lytic replication, Catcher peptides redistribute to assembling capsids, creating the appearance of fluorescent foci (e.g., see Fig. 2a in the main text). Therefore, imaging the cell biological process of lytic replication using Phollow requires relatively high expression of Catcher peptides to adequately label virions, but not so high that the signal of viral foci is obscured by the background fluorescence from unbound Catcher peptides. In our studies, we tried multiple inducible and constitutive promoters for Catcher peptide expression.

Ultimately, through trial and error, we identified a constitutive promoter that produced an adequate balance of labeling and background. It is likely that the expression of fluorescent Catcher peptides will need to be tuned for each phage and bacterial host cell.

Phollow Phage Characterization (Fig. 2)

Once a Phollow phage is constructed, it is important to probe whether Phollow tagging may have disrupted any virion activities or traits; namely, replication dynamics, cell biology, and virion morphology.

Replication dynamics

The induction and release of infectious particles are crucial features of a phage's life cycle. Confirming that Phollow phage infectivity is comparable to an unmodified wild-type phage can be done using conventional plaque- or lysogen-forming unit (PFU or LFU) assays (e.g., as in Fig. 1d of the main text). Moreover, visualizing adsorption of Phollow phage virions to target bacterial cells can help confirm that fluorescent viral-like particles are in fact virions (e.g., as in Fig. 5c of the main text). Lastly, if an adsorption experiment is done using a target cell expressing a fluorescent Catcher protein (e.g., as in Fig. 5d–g of the main text), then the ability of a Phollow phage to replicate and spread can also be confirmed.

Fig 2. Phollow phage characterization

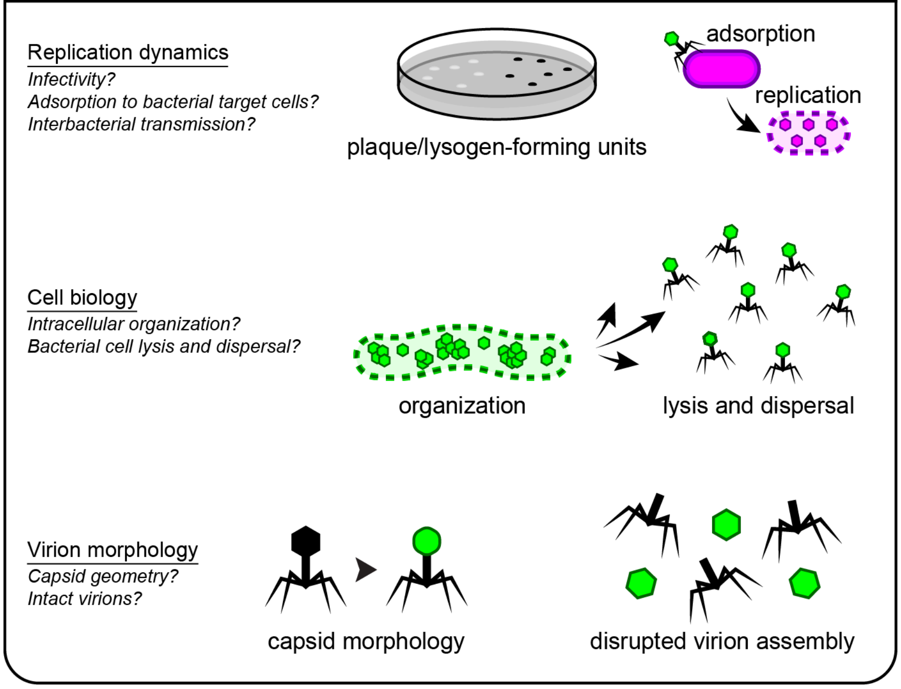

Cell biology

The intracellular organization and dynamics of viral particles during lytic replication are largely unexplored for many phages. However, observing the assembly and dispersal of fluorescently marked Phollow phages during lytic replication can help evaluate their overall functionality. For example, as depicted in Fig. 2a,l in the main text, following the ontogeny of DuoHS Phollow phage virions revealed that they assemble as large multi-virion aggregates that then rapidly disassemble into individual particles upon cell lysis. Observing this process helped connect the large virion structures observed inside cells to infectious particles outside of cells. Moreover, charting intracellular virion dynamics informs the use of other techniques, like imaging flow cytometry, that are able to further quantify features of lytic replication.

Virion morphology

Another aspect of Phollow phage virions that should be considered is whether the tagging process created any morphological abnormalities, which may hint at a possible reduction in virion integrity. Expansion microscopy and transmission electron microscopy (e.g., as in Fig. 2g–k and Extended Data Fig. 5 of the main text) can be used to inspect structural features of capsids as well as the frequency of intact capsid/tail assemblages.

Experimental Considerations

Once a Phollow phage is constructed and its basic features are characterized, there are some additional considerations for incorporating it into experimental schemes. Here we outline some of the lessons learned during the development of Phollow that may help other researchers employ the Phollow system in their own work.

Live imaging of Phollow phages

It is important to consider the spatial and temporal constraints that are inherently associated with imaging nanoscopic viral particles in the context of microscopic bacterial host cells and macroscopic animal tissues. Phage virions are exceptionally small and thus, even if they are abundant within a sample, it may still be incredibly difficult to locate them within a single field of view. Moreover, the relative brightness of Phollow phage virions is low compared to surrounding bacterial cells and other labeled structures such as animal tissues. Therefore, processing images containing Phollow phage virions often requires specialized lookup tables (LUTs) for pseudocoloring images to enhance visual contrast. In some instances it is also necessary to mask different regions or objects so that virions can be sufficiently distinguished within complex environments like the zebrafish gut (e.g., as in various images in Fig. 4 of the main text). For the current work, we created several custom LUTs that can be used with ImageJ and are provided as supplemental files.

Phage induction, replication, and spread are also highly dynamic processes, which means that visualizing phage virions greatly depends on when they are imaged. For example, while following lytic replication over time in standard test tube-based experiments, we found that Phollow phage virions eventually decay and become difficult to image at later time points. We surmise that the massive cell lysis event during lytic replication under these conditions—which likely releases proteases, reactive oxygen species, and other damaging molecules—leads to virion destruction. The importance of timing was also revealed during the imaging of phage outbreaks within the zebrafish gut (as in Fig. 4f on the main text). We found that Phollow phage virions were highly abundant shortly after antibiotic-mediated induction, but that at later time points they had completely vanished (likely being degraded, translocated to other tissues, and expelled from the gut).Therefore, we recommend probing multiple time windows when studying replication dynamics using Phollow.

Flow cytometry/virometry with Phollow phages

Fluorescent Phollow phages are amenable to both imaging flow cytometry and flow virometry methods. The application of imaging flow cytometry to quantify bacterial cells harboring lytically replicating phage is fairly straightforward but it requires that the intracellular organization of assembling virions be distinguishable from non-induced cells. In the current work, we visually inspected individual cell events to manually curate gates using the built-in machine learning software of the ImageStreamX MKII cytometer. As a starting point for other researchers, we have provided the template files containing the gating algorithms we generated as supplemental files.

A crucial consideration for flow virometry is the degree to which the signal to noise ratio can be controlled. Fluorescent cell debris, the autofluorescent properties of culture media, and spectral overlap in multiplexed (i.e., mixed virion) samples can significantly interfere with the detection of Phollow phages (as we described in the main text and experimentally addressed in Fig. 3b,c). Although we found that the incorporation of virion purification steps helped to address this problem, they do not fully remove all of the fluorescent background. This is, in part, because of the exquisite sensitivity of spectral flow cytometry instruments. We demonstrated in Fig. 3 of the main text that flow virometry is feasible with Phollow phages but there remains some notable constraints. Namely, the limited availability of genetically encoded fluorescent proteins with sufficiently narrow excitation/emission spectra and brightnesses ultimately impedes efforts to enhance the signal to noise ratio within some samples.

A powerful utility of spectral flow cytometry is that it makes it possible to analyze multiplexed samples containing numerous distinctly labeled Phollow phage virions (e.g., as in Fig. 3e of the main text). However, in well-mixed test tube-based experiments where there is active and widespread lytic replication in multiple bacterial cell populations, we found that virion cross-labeling can occur. That is, as cells lyse, fluorescent Catcher peptides that are released are able to bind free SpyTag positions on virions originating from cells of a different color. This apparent cross-labeling appears to not occur as much within the spatially structured environment of the zebrafish gut and is not as intense during short-term experiments. In most cases where cross-labeling occurs, the signal of the original tag dominates and is easily distinguished by microscopy, whereas any level of cross-labeling can exacerbate the initial problem of signal to noise ratio during flow virometry-based experiments.
