# Supplementary material for: Phollow reveals in situ phage transmission dynamics in the zebrafish gut microbiome at single-virion resolution
Source: Nat Microbiol. 2025 Apr 18;10(5):1067–83. doi: 10.1038/s41564-025-01981-1 (PMC12055606; doi:10.1038/s41564-025-01981-1)
Supplement: Supplementary file 2 — Reporting Summary [file 41564_2025_1981_MOESM2_ESM.pdf]

nature portfolio

Corresponding author(s): Travis Wiles

Last updated by author(s): Feb 24, 2025

Reporting Summary

Nature Portfolio wishes to improve the reproducibility of the work that we publish. This form provides structure for consistency and transparency in reporting. For further information on Nature Portfolio policies, see our Editorial Policies and the Editorial Policy Checklist.

Statistics

For all statistical analyses, confirm that the following items are present in the figure legend, table legend, main text, or Methods section.

n/a	Confirmed	
☐	☒	The exact sample size (*n*) for each experimental group/condition, given as a discrete number and unit of measurement
☐	☒	A statement on whether measurements were taken from distinct samples or whether the same sample was measured repeatedly
☐	☒	The statistical test(s) used AND whether they are one- or two-sided<br>*Only common tests should be described solely by name; describe more complex techniques in the Methods section.*
☐	☒	A description of all covariates tested
☐	☒	A description of any assumptions or corrections, such as tests of normality and adjustment for multiple comparisons
☐	☒	A full description of the statistical parameters including central tendency (e.g. means) or other basic estimates (e.g. regression coefficient) AND variation (e.g. standard deviation) or associated estimates of uncertainty (e.g. confidence intervals)
☐	☒	For null hypothesis testing, the test statistic (e.g. *F*, *t*, *r*) with confidence intervals, effect sizes, degrees of freedom and *P* value noted<br>*Give P values as exact values whenever suitable.*
☒	☐	For Bayesian analysis, information on the choice of priors and Markov chain Monte Carlo settings
☒	☐	For hierarchical and complex designs, identification of the appropriate level for tests and full reporting of outcomes
☒	☐	Estimates of effect sizes (e.g. Cohen's *d*, Pearson's *r*), indicating how they were calculated

Our web collection on statistics for biologists contains articles on many of the points above.

Software and code

Policy information about availability of computer code

Data collection	Omega version 5.7 R2 was used to collect data regarding bacterial growth and lysis curves.<br>Agilent NovoExpress version 1.5.6 was used to collect flow cytometry data<br>INSPIRE ISX version 200.1.681.0 was used to acquire imaging flow cytometry data<br>SpectroFlow v2 was used to collect flow virometry data<br>ECHO Pro version 6.4.2 for the acquisition of fluorescent microscopy images<br>ZEN Black 2.3 for super resolution microscopy images acquired with Elyra 7 microscope<br>ZEN blue 3.2 for super resolution microscopy images acquired with LSM 980 Airyscan 2.0 microscope<br>Gatan Microscopy Suite version 3.2.1461.0 was used for transmission electron microscopy
Data analysis	GraphPad Prism 6 was used to graph data and perform statistical analyses.<br>FCS Express 7.22.0031 was used to analyze flow virometry data<br>FlowJo 10.10 was used to analyze flow cytometry data<br>IDEAS 6.2 was used to analyze imaging flow cytometry data<br>ZEN Black 2.3 with SIM2 was used to reconstruct super resolution images acquired with Elyra 7 microscpe<br>ZEB blue 3.2 was used to reconstruct super resolution images acquired with LSM 980 Airyscan 2.0 microscope<br>FIJI (ImageJ) 2.14/1.54f was used for image processing and analysis.<br>Imaris 10.1.1 was used to generate three-dimensional renderings from microscopy images.<br>Pharokka (v1.7.1) was employed to annotate the P2-like prophage genomes of DuoHS (from E. coli HS) and DuoZ11 (from Plesiomonas ZOR0011), utilizing the -g 'prodigal' flag as the gene caller.

Genome visualization was performed using pyGenomeViz (v1.4.1) with MMseq to illustrate coding sequence homology

For manuscripts utilizing custom algorithms or software that are central to the research but not yet described in published literature, software must be made available to editors and reviewers. We strongly encourage code deposition in a community repository (e.g. GitHub). See the Nature Portfolio guidelines for submitting code & software for further information.

Data

Policy information about availability of data

All manuscripts must include a data availability statement. This statement should provide the following information, where applicable:

- Accession codes, unique identifiers, or web links for publicly available datasets
- A description of any restrictions on data availability
- For clinical datasets or third party data, please ensure that the statement adheres to our policy

All numerical data underlying plots shown in main and extended data figures are provided in Supplementary Data 4. Image source data files were deposited in Figshare (https://figshare.com/s/50dd81a7714a89f05ea0).

Research involving human participants, their data, or biological material

Policy information about studies with human participants or human data. See also policy information about sex, gender (identity/presentation), and sexual orientation and race, ethnicity and racism.

Reporting on sex and gender	N/A
Reporting on race, ethnicity, or other socially relevant groupings	N/A
Population characteristics	N/A
Recruitment	N/A
Ethics oversight	N/A

Note that full information on the approval of the study protocol must also be provided in the manuscript.

Field-specific reporting

Please select the one below that is the best fit for your research. If you are not sure, read the appropriate sections before making your selection.

☒ Life sciences ☐ Behavioural & social sciences ☐ Ecological, evolutionary & environmental sciences

For a reference copy of the document with all sections, see nature.com/documents/nr-reporting-summary-flat.pdf

Life sciences study design

All studies must disclose on these points even when the disclosure is negative.

Sample size	The sample sizes for in vitro experiments were first determined empirically based on pilot studies which allowed us to then assess data variability and determine the necessary sample sizes to determine statistical differences. For in vivo experiments, we based our sample size on prior published research (doi:10.1371/journal.pbio.1002517, doi:10.1371/journal.pbio.3000661, doi:10.1128/mbio.01877-18, doi:10.1371/journal.pbio.2000689, doi:10.1016/j.chom.2015.10.009,doi: 10.1073/pnas.1907567116) and power analyses, concluding that 15-25 fish total are typically sufficient to ensure adequate statistical power.
Data exclusions	Pilot and optimization experiments were excluded from the final graphed data. In some instances, experimental data were excluded due to issues such as the decay of drug potency (e.g., mitomycin C), or issues that can sometimes arise with germ-free zebrafish derivation (e.g., bacterial contamination or poor larval development/health). Any data that were excluded was done so based on our best judgment.
Replication	Reproducibility was assessed through pilot experiments and confirmed by conducting the final optimized experimental schemes with sufficient sample sizes and at least 3 biological replicates.
Randomization	This study aimed to observe and describe phage outbreak dynamics under highly controlled conditions. The controlled setup effectively reduces bias and ensures reproducibility, making randomization unnecessary for achieving the study's objectives.
Blinding	Given the descriptive nature of this study, blinding was not applicable. The primary outcomes here reported were based on direct observations and quantitative analyses using automated and standardized protocols, which minimized the risk of bias.

Reporting for specific materials, systems and methods

We require information from authors about some types of materials, experimental systems and methods used in many studies. Here, indicate whether each material, system or method listed is relevant to your study. If you are not sure if a list item applies to your research, read the appropriate section before selecting a response.

Materials & experimental systems

n/a	Involved in the study
☒	☐ Antibodies
☒	☐ Eukaryotic cell lines
☒	☐ Palaeontology and archaeology
☐	☒ Animals and other organisms
☒	☐ Clinical data
☒	☐ Dual use research of concern
☒	☐ Plants

Methods

n/a	Involved in the study
☒	☐ ChIP-seq
☐	☒ Flow cytometry
☒	☐ MRI-based neuroimaging

Animals and other research organisms

Policy information about studies involving animals; ARRIVE guidelines recommended for reporting animal research, and Sex and Gender in Research

Laboratory animals	Danio rerio lines AB and TgBAC(nkx2.2a:megfp). ". All zebrafish used in this study were larvae, between the ages of 4- and 7-days post fertilization.
Wild animals	No wild animals were used in the study.
Reporting on sex	Zebrafish used in this study were larvae, between the ages of 4- and 7-days post fertilization. Sex differentiation occurs later in zebrafish development and thus was not a factor in our experiments.
Field-collected samples	No field collected samples were used in the study.
Ethics oversight	All experiments with zebrafish were done in accordance with protocols approved by the University of California, Irvine Institutional Animal Care and Use Committee (protocol #AUP-23-126).

Note that full information on the approval of the study protocol must also be provided in the manuscript.

Plants

Seed stocks	*Report on the source of all seed stocks or other plant material used. If applicable, state the seed stock centre and catalogue number. If plant specimens were collected from the field, describe the collection location, date and sampling procedures.*
Novel plant genotypes	*Describe the methods by which all novel plant genotypes were produced. This includes those generated by transgenic approaches, gene editing, chemical/radiation-based mutagenesis and hybridization. For transgenic lines, describe the transformation method, the number of independent lines analyzed and the generation upon which experiments were performed. For gene-edited lines, describe the editor used, the endogenous sequence targeted for editing, the targeting guide RNA sequence (if applicable) and how the editor was applied.*
Authentication	*Describe any authentication procedures for each seed stock used or novel genotype generated. Describe any experiments used to assess the effect of a mutation and, where applicable, how potential secondary effects (e.g. second site T-DNA insertions, mosiacism, off-target gene editing) were examined.*

Flow Cytometry

Plots

Confirm that:

☒ The axis labels state the marker and fluorochrome used (e.g. CD4-FITC).

☒ The axis scales are clearly visible. Include numbers along axes only for bottom left plot of group (a 'group' is an analysis of identical markers).

☒ All plots are contour plots with outliers or pseudocolor plots.

☒ A numerical value for number of cells or percentage (with statistics) is provided.

Methodology

Sample preparation	Flow virometry: Samples were collected from 1 mL of bacterial culture and treated with chloroform (0.1x culture volume) for 5 minutes. After centrifugation, the aqueous layer was collected and treated with DNAse, 20 mN EDTA and 1M NaCl as described in materials and methods. The supernatant was used for flow virometry.<br>Flow cytometry and imaging flow cytometry: Bacterial samples were fixed in 4% paraformaldehyde solution for 30 minutes at room temperature. Samples suspended in 0.7% saline solution were stored at 4°C until further analysis.
Instrument	Flow virometry: Cytek's Northern Lights 3-laser spectral flow cytometer or Cytek's Aurora 5-laser spectral flow cytometer.<br>Flow cytometry: Novocyte flow cytometer (Agilent). Imaging flow cytometry: mageStreamX MKII cytometer (Cytek Biosciences).
Software	Flow virometry collection: SpectroFlow version 2. Flow virometry analysis: FCS Express 7.22.0031. Flow cytometry collection: Agilent NovoExpress version 1.5.6. Flow cytometry analysis: FlowJo 10.10. Imaging flow cytometry collection: INSPIRE ISX. Imaging flow cytometry analysis: IDEAS 6.2
Cell population abundance	For all cases, abundance of the relevant population was dependent on antibiotic induction efficiency.<br>Flow virometry: Purity of sample was determined using Sperotech's 0.13 µm yellow sizing beads and fluorescent microscopy as comparison.<br>Imaging flow cytometry: Purity of the sample was determined by visual assessment of gated events.
Gating strategy	Flow virometry: Phage particle-sized events were first identified and detected on a FSC-H vs SSC-H plot using Spherotech's 0.13 µm yellow sizing beads (NFPPS-0152-5) while adjusting voltage gains for FSC and SSC. A total of 10,000-50,000 events were captured depending on the phage availability in the samples. Raw FCS files were unmixed with reference controls of each respective Phollow phage sample containing the SpyTag and SpyCatcher, and the unstained reference control being a P2 phage deletion mutant strain.<br>Unmixed FCS files were analyzed using FCS Express. Phages were gated based on their size relative to the 100 µm sizing beads described above. Single particle gating on SSC-A vs SSC-H was used to discriminate against doublets. Positive MFI gates were drawn for each respective fluorescent Catcher peptide using samples negative for the SpyTag but containing the fluorescent Catcher peptide.<br><br>Flow cytometry: Cells were first identified based on their forward (>1,000) and side scatter profile and subsequently analyzed for mNG signal. Gates to identify SOS positive events were determined using an untagged wild-type E. coli HS strain as a negative control, and an E. coli HS strain.<br><br>Flow imaging flow cytometry: Samples were gated based on pixel intensity variance and contrast morphology features generated with the machine learning algorithm included in IDEAS software. Gates were manually curated and adjusted by visually inspecting and excluding inappropriate events

☒ Tick this box to confirm that a figure exemplifying the gating strategy is provided in the Supplementary Information.
